# Cryo-EM structure of the complete inner kinetochore of the budding yeast point centromere

**DOI:** 10.1126/sciadv.adg7480

**Published:** 2023-07-28

**Authors:** Tom Dendooven, Ziguo Zhang, Jing Yang, Stephen H. McLaughlin, Johannes Schwab, Sjors H. W. Scheres, Stanislau Yatskevich, David Barford

**Affiliations:** MRC Laboratory of Molecular Biology, Francis Crick Avenue, Cambridge CB2 0QH, UK.

## Abstract

The point centromere of budding yeast specifies assembly of the large kinetochore complex to mediate chromatid segregation. Kinetochores comprise the centromere-associated inner kinetochore (CCAN) complex and the microtubule-binding outer kinetochore KNL1-MIS12-NDC80 (KMN) network. The budding yeast inner kinetochore also contains the DNA binding centromere-binding factor 1 (CBF1) and CBF3 complexes. We determined the cryo–electron microscopy structure of the yeast inner kinetochore assembled onto the centromere-specific centromere protein A nucleosomes (CENP-A^Nuc^). This revealed a central CENP-A^Nuc^ with extensively unwrapped DNA ends. These free DNA duplexes bind two CCAN protomers, one of which entraps DNA topologically, positioned on the centromere DNA element I (CDEI) motif by CBF1. The two CCAN protomers are linked through CBF3 forming an arch-like configuration. With a structural mechanism for how CENP-A^Nuc^ can also be linked to KMN involving only CENP-QU, we present a model for inner kinetochore assembly onto a point centromere and how it organizes the outer kinetochore for chromosome attachment to the mitotic spindle.

## Teaser

Conserved sequence motifs of yeast point centromeres orchestrate assembly of the inner kinetochore onto a CENP-A nucleosome.

## INTRODUCTION

Kinetochores are critical to the faithful inheritance of genetic information and function by attaching sister chromatids to the mitotic spindle and by harnessing the power of microtubule depolymerization to move them to the spindle poles (*1*–*3*). In many organisms, kinetochore assembly is restricted to the centromere, a specialized region of chromatin defined by nucleosomes containing the histone H3 variant CENP-A (CENP-A^Nuc^) (*4*–*6*). Kinetochores are structurally and functionally delineated into the inner and outer kinetochore. The inner kinetochore centromere-associated inner kinetochore (CCAN) complex (14 to 16 subunits; table S1) associates with the centromere, generally through specific recognition of CENP-A^Nuc^. CCAN then connects the centromere to the outer kinetochore, the 10-subunit KNL1-MIS12-NDC80 (KMN) network. Ndc80c of this network, in association with either the Dam1/DASH complex in yeast or the Ska complex in humans, attaches kinetochores to spindle microtubules.

The point centromeres of budding yeast and regional centromeres of higher eukaryotes differ substantially in size and higher-order structure but, nevertheless, share a conserved underlying architecture. Point centromeres comprise an individual CENP-A^Nuc^–kinetochore complex that attaches to a single microtubule (*7*, *8*). The budding yeast centromere is genetically defined by a ~120–base pair (bp) sequence that is sufficient to template complete mitotic and meiotic centromere function (*9*), and onto which CENP-A^Nuc^ is perfectly positioned (*10*–*12*). All 16 *Saccharomyces cerevisiae* centromeres comprise three centromere DNA elements (CDEs) (fig. S1A) (*13*–*15*). The CDEI and CDEIII motifs are highly conserved and function to bind two protein complexes, CBF1 and CBF3, specific to the point centromere kinetochores of budding yeast (*16*–*19*). Whereas CDEIII and CBF3 are essential for viability (*19*–*26*), cells with CDEI disrupted remain viable but exhibit mitotic chromosome loss, and in meiosis I, defective centromere function (*20*, *23*, *27*–*29*). CDEII is less well conserved; however, its AT-rich DNA sequence is proposed to be favorable for CENP-A^Nuc^ assembly in vivo because of its increased tendency to curve (*30*–*33*).

We previously determined a cryo–electron microscopy (cryo-EM) structure of *S. cerevisiae* CCAN in complex with CENP-A^Nuc^ reconstituted with non-native Widom 601 DNA (W601) (*34*). In this cryo-EM reconstruction, we observed a single CCAN protomer assembled onto CENP-A^Nuc^. The structure delineated the overall architecture of CCAN and revealed an interaction between the unwrapped DNA terminus of CENP-A^Nuc^ and a deep positively charged DNA binding channel situated at the center of a single CCAN protomer. CENP-A^Nuc^ comprising native centromeric (*CEN*) DNA (*CEN*-CENP-A^Nuc^) on the other hand may have different DNA wrapping properties from that reconstituted with the W601 sequence. In addition, the binding of the inner kinetochore complexes CBF1 and CBF3 to specific sequence elements present in *CEN*-CENP-A^Nuc^ but not in *W601*-CENP-A^Nuc^ may influence the organization and stoichiometry of CCAN protomers on CENP-A^Nuc^. In this study, to understand how conserved sequence motifs of yeast point centromeres orchestrate assembly of the inner kinetochore onto CENP-A^Nuc^, we determined the cryo-EM structure of the inner kinetochore–point centromere complex. We used a near-native centromere sequence (termed *C0N3*) incorporating the CBF1- and CBF3-binding elements CDEI and CDEIII, respectively, with stabilizing W601 DNA substituted for CDEII (fig. S1A), and provided in vivo support for our models. Both CBF1 and CBF3 function to organize two CCAN protomers onto a central CENP-A^Nuc^. Dimeric CBF1 binds CDEI with its basic helix-loop-helix (bHLH) segments to position one of the two CCAN protomers 5′ of CENP-A^Nuc^. We describe an alternative DNA binding mode for this CCAN where CBF1 assists in the topological entrapment of DNA via the CENP-HIK^Head^-TW module of CCAN. A second CCAN assembles onto the 3′ end of the DNA using a nontopological DNA binding mode identical to our previous CCAN:*W601*-CENP-A^Nuc^ structure (*34*, *35*), generating an asymmetric, dimeric CCAN inner kinetochore. The two CCAN modules are bridged by CBF3^Core^, now displaced from the CENP-A^Nuc^ face (*36*), to fulfill a stabilizing role at the kinetochore. Together, the inner kinetochore forms an arch-like structure around a central CENP-A^Nuc^, embedding ~150 bp of centromeric DNA.

We also present a structural explanation for how the CENP-A N terminus (CENP-A^N^) interacts with CENP-QU (*37*–*40*). Unexpectedly, the CENP-A^N^ binding site on CENP-QU is autoinhibited in the context of the assembled CCAN. Thus, CENP-QU binds CENP-A^N^ independently of CCAN, suggesting a separate CENP-A^Nuc^-CENP-QU connection to the outer kinetochore.

## RESULTS

### The inner kinetochore comprises two CCAN protomers positioned on a central CENP-A^Nuc^ by the CBF1 and CBF3 complexes

We overcame the inherent instability of CENP-A^Nuc^ reconstituted with native centromeric DNA (*36*, *41*) by using a chimeric 153 bp DNA sequence (*C0N3*). The design of *C0N3* was guided by the cryo-EM structure of the *S. cerevisiae* centromeric nucleosome (*CEN3*-CENP-A^Nuc^) stabilized by a single-chain antibody fragment of the variable region of the light and heavy chains (scFv) (*36*). This structure defined the position of *CEN3* DNA on the histone octamer and revealed that a 20 bp palindrome in *CEN3* is centered exactly on the dyad axis of the histone octamer (fig. S1A). Using this information, we designed *C0N3* incorporating the CDEI and CDEIII elements, as well as their flanking sequences, and substituted W601 sequence for most of CDEII. A three-dimensional (3D)–based alignment of *CEN3*-CENP-A^Nuc^ (*36*) and *W601*-CENP-A^Nuc^ [Protein Data Bank (PDB): 7ON1] was used to define the region of W601 DNA to substitute for CDEII of *CEN3* (fig. S1A). *C0N3* was used to generate a native-like but more stable CENP-A^Nuc^ (*C0N3*-CENP-A^Nuc^) for reconstituting the holo–inner kinetochore complex comprising CCAN, CBF1, and CBF3^Core^ (CBF1:CCAN:*C0N3*-CENP-A^Nuc^:CBF3^Core^) (fig. S1, B and C, and table S1). Size exclusion chromatography–multiangle light scattering (SEC-MALS) analysis showed that the holo–inner kinetochore complex had an overall molecular mass of 1.6 MDa, consistent with a stoichiometry of (CBF1)_2_:(CCAN)_2_:*C0N3*-CENP-A^Nuc^:CBF3^Core^ (fig. S1D and table S1). The holo–inner kinetochore complex reconstituted with entirely native centromeric DNA (153 bp *CEN3*; fig. S1A) had an identical mass of 1.61 MDa (fig. S1, E to G). Thus, holo–inner kinetochore complexes reconstituted using either native *CEN3* or *C0N3* DNA share identical compositions matching the expected molecular mass of 1.61 MDa (fig. S1, D and G; table S1).

**Fig. 1.**
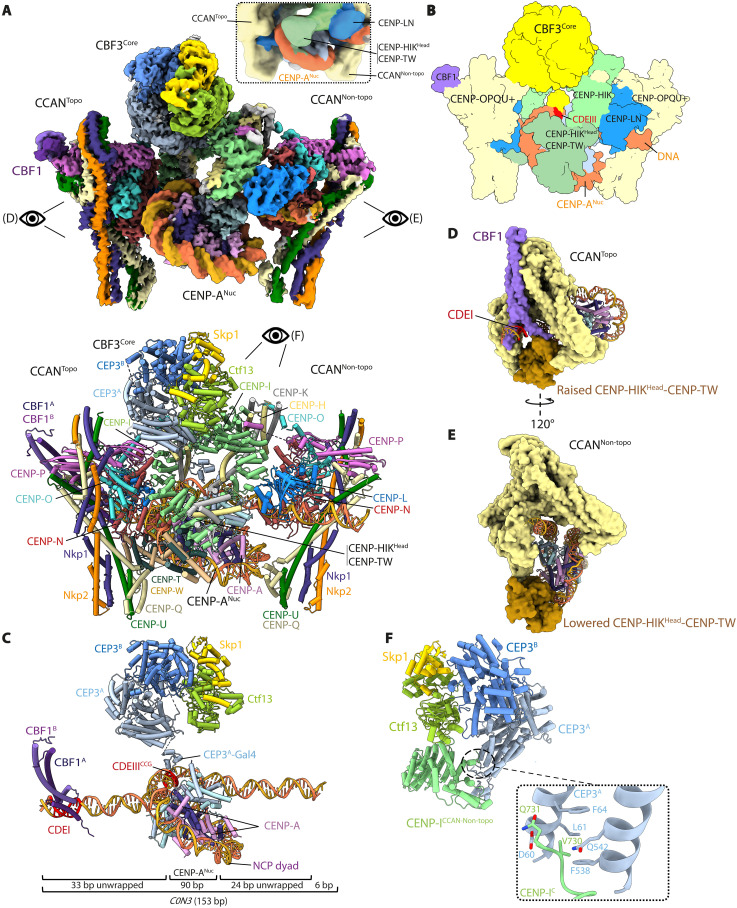
The inner kinetochore comprises two CCAN protomers bound to a central CENP-A^Nuc^ organized by the CBF1 and CBF3 complexes. (**A**) Composite cryo-EM map of the complex (top) and annotated ribbons representation (below). Two CCAN protomers flank the central CENP-A^Nuc^ in an asymmetric arrangement. CCAN^Non-topo^ engages the 3′ end of the *C0N3* DNA in an open configuration, similar to (*34*). CCAN^Topo^ engages the 5′ end of *C0N3*-CENP-A^Nuc^ and topologically entraps the DNA together with CBFI. CBF3^Core^ bridges the two CCAN modules. The single scFv bound to CENP-A^Nuc^ is not visible in this view (shown in fig. S2C). Inset: A portion of the consensus cryo-EM (fig. S3A) map at a lower contour level to show EM density for the CENP-HIK^Head^-TW module. (**B**) Schematic of the complex. (**C**) CBF1 and CBF3^Core^ interact with CDEI and CDEIII, respectively. CDEI is located within the 5′ unwrapped DNA duplex of CENP-A^Nuc^, whereas CDEIII is located within the CENP-A^Nuc^ DNA gyre (SHL4). The body of CBF3^Core^ is distal to the face of the CENP-A nucleosome, a configuration that is different from (*36*), where the CBF3^Core^ sits proximal to the nucleosome (fig. S4, C and D). The 153 bp *C0N3* DNA is indicated: A total of 90 bp of DNA wraps the CENP-A^Nuc^ gyre, with 33 and 24 bp unwrapped at the 5′ and 3′ ends, respectively. (**D** and **E**) Views of CCAN^Topo^ (D) and CBF1:CCAN^Non-t^^opo^ (E) in surface representation showing their different modes of binding CENP-A^Nuc^. (**F**) CEP3^A^ of CBF3 forms extensive contacts with the C-terminal region of CENP-I of CCAN^Non-topo^ (movie S1).

For cryo-EM analysis, we took advantage of the CENP-A^Nuc^–stabilizing scFv (fig. S1, H and I) (*36*). Because the binding site for scFv on H2A-H2B (*36*) overlaps with the CENP-C binding site (*34*), we omitted CENP-C from our structural analysis (CCAN^ΔC^). This CBF1:CCAN^ΔC^:CENP-A^Nuc^:CBF3^Core^:scFv complex is referred to as the inner kinetochore [CBF1:CCAN^ΔC^:*C0N3*-CENP-A^Nuc^:CBF3^Core^:scFv (IK*^C0N3^*)]. A consensus 3D reconstruction of the complex was limited to ~5 Å resolution because of conformational heterogeneity (fig. S2, A and B; fig. S3A; and table S2); however, multibody refinement of rigid domains extended the resolution to 3.7 to 3.8 Å (Fig. 1, A and B, and fig. S3, A and B). In this reconstruction, we observed two CCAN protomers, CENP-A^Nuc^, a CBF1 homodimer, CBF3^Core^, and one scFv (Fig. 1, fig. S2C, and movie S1), in agreement with the composition and stoichiometry of components constituting the holo–inner kinetochore complex (including CENP-C) determined using SEC-MALS (fig. S1D and table S1). To test whether BS3 cross-linking affected the overall structure of IK*^C0N3^*, we collected a negative-stain EM dataset of non–cross-linked IK*^C0N3^*. The resultant 2D class averages matched calculated 2D projections of the cross-linked IK*^C0N3^* cryo-EM maps, as well as the corresponding experimental 2D class averages (fig. S2B). This indicates that the cross-linked cryo-EM IK*^C0N3^* structure is representative of the non–cross-linked state.

In the inner kinetochore complex, CENP-A^Nuc^ is wrapped by only one turn of DNA (~90 bp), in a left-handed configuration (Fig. 1, A to C). Both the 5′ and 3′ DNA ends of CENP-A^Nuc^ are therefore unwrapped, albeit to different extents: 33 bp and 24 bp at the 5′ and 3′ ends, respectively (Fig. 1C and fig. S4, A and B). The two unwrapped DNA duplexes create binding sites for two asymmetrically arranged CCAN protomers, termed CCAN^Topo^ and CCAN^Non-topo^, which bind CENP-A^Nuc^ through two different binding modes (Fig. 1, A to E). These two modes of CCAN binding to centromeric DNA differ in the position of the CENP-HIK^Head^-TW module, constituting topological and nontopological DNA binding mechanisms (CCAN^Topo^ and CCAN^Non-topo^) (Fig. 1, D to E). Together, the two CCAN protomers form an arch-like structure around CENP-A^Nuc^, bridged by CBF3^Core^, which embeds a total of 150 bp of DNA (Fig. 1, A to C).

**Fig. 2.**
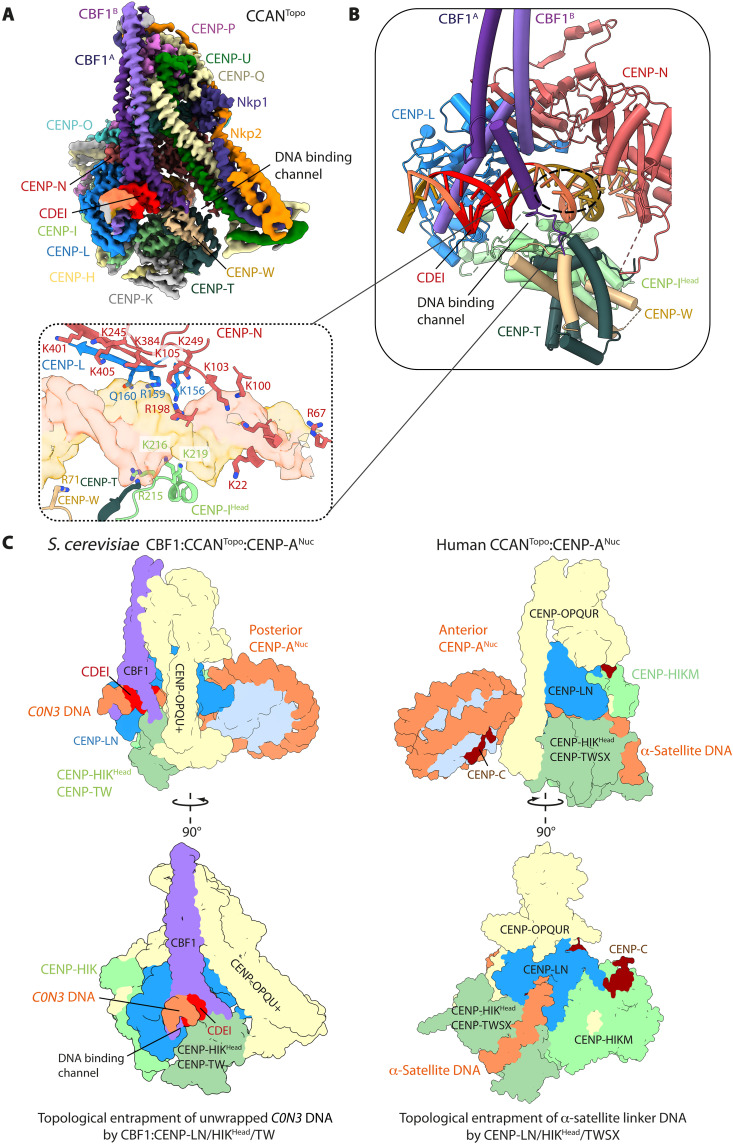
Topological entrapment of *C0N3* DNA by CCAN^Topo^. (**A**) Cryo-EM map of the CBF1:CCAN complex bound to a 30 bp DNA segment of *C0N3* containing CDEI, at 3.4 Å. (**B**) Two subunits of CBF1 interact through their basic α helices with the major groove of the *C0N3* DNA at the entry of the CENP-LN-HIK^Head^-TW channel . Inset: CENP-LN, CENP-I^Head^, and CENP-TW form a basic, closed chamber that topologically entraps DNA. (**C**) Comparison of the *S. cerevisiae* and human CCAN^Topo^-CENP-A^Nuc^ (*42*) modules. *S. cerevisiae* CCAN topologically entraps the unwrapped *C0N3* DNA, 5′ of CENP-A^Nuc^, in its basic CBF1:CENP-LN-HIK^Head^-TW channel. Human CCAN entraps α-satellite linker DNA in its CENP-LN-HIK^Head^-TWSX channel, wrapping the DNA around the CENP-TW histone fold domains.

The organization of CBF1:CCAN^Topo^ at the 5′ end of *C0N3*-CENP-A^Nuc^ is identical to that observed for an individual CBF1:CCAN complex bound to *C0N3* DNA determined at a resolution of 3.4 Å (Fig. 2A; figs. S2, D and E, and S3C; and table S2). One consequence of CBF1 engaging CCAN is that the CENP-LN channel is extended, so a total of 25 bp of DNA interact with CBF1:CCAN (Fig. 2B). In addition, the CENP-LN channel converts into an enclosed basic chamber that completely surrounds the DNA duplex, a configuration notably reminiscent of how human CCAN grips the linker DNA of an α-satellite-CENP-A^Nuc^ (Fig. 2C) (*42*). In the latter, the linker DNA is partially wrapped around the CENP-TW histone fold domains, a feature that might be specific for regional centromeres and is not conserved in the topologically entrapped DNA of the budding yeast inner kinetochore (Fig. 2C). Furthermore, unlike human CCAN, CENP-SX does not assemble onto CCAN in budding yeast, as observed by us and others (*43*). While CBF1 positions CCAN at the CDEI motif of budding yeast point centromeres, it is unknown how and to what extent its functional analog CENP-B recruits human CCAN to B-box motifs at regional centromeres.

**Fig. 3.**
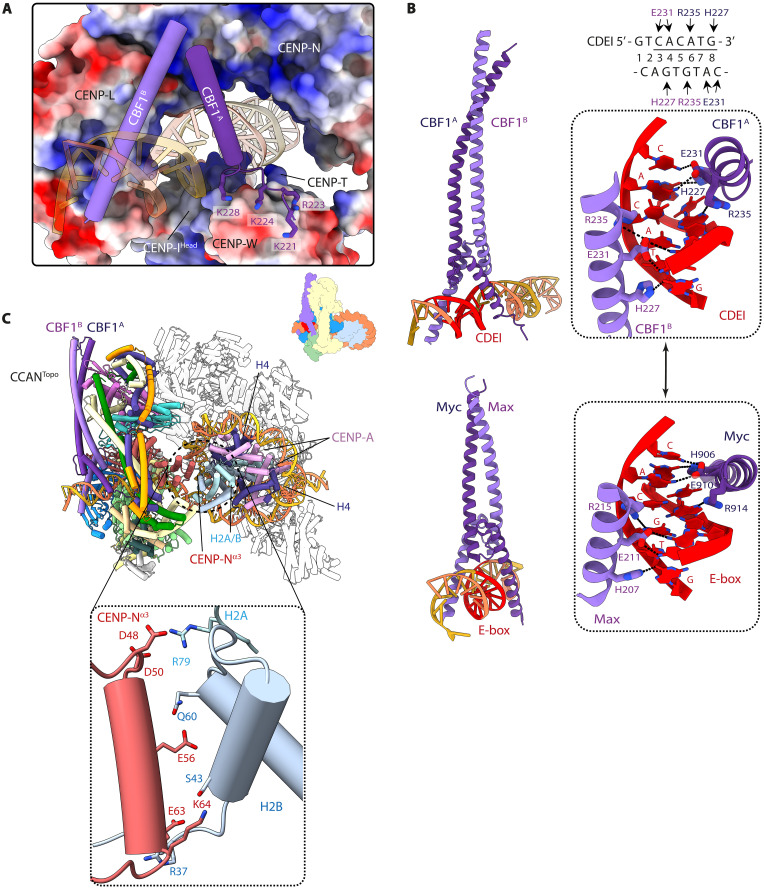
CBF1 extends the basic DNA binding CENP-LN channel through interactions with CDEI. (**A**) The DNA binding tunnel of CCAN has a marked electropositive potential and is extended by the basic α helices of CBF1. An acidic patch on CENP-TW binds basic residues of the CBF1^A^ helix, thereby unfolding the N-terminal half of the helix. (**B**) The sequence-specific contacts of CBF1 with CAC(A/G)TG of CDEI (top) are nearly identical to how the Myc-Max transcription factor interacts with its cognate E-box CACGTC motif (bottom). (**C**) Details of how the CENP-N α3 helix interacts with basic residues of histones H2A-H2B that are exposed because of unwrapping of the CENP-A^Nuc^ DNA gyre. Hence, 33 bp of *C0N3*-CENP-A^Nuc^ is unwrapped at its 5′ end.

Formation of the enclosed DNA binding chamber of the budding yeast CCAN results from the mobile CENP-HIK^Head^-TW module adopting a raised position to directly contact the DNA duplex (Figs. 2, A and B, and 3A; and fig. S5A). Specifically, a basic surface on CENP-I^Head^ forms extensive contacts with the DNA-phosphate backbone. This topologically enclosed DNA binding chamber is further stabilized through interactions between an acidic patch on CENP-TW with the CBF1^A^ protomer (Fig. 3A). The bHLH leucine zipper domain of CBF1 is the only region of CBF1 observed in the cryo-EM map. The CBF1 homodimeric leucine zipper coiled coil interacts with the back-face of CCAN (fig. S6A), forming a hydrophobic interface with CENP-Q, centered on CENP-Q^Ile292^ (fig. S6A, inset b). This agrees with the observation that truncation and deletion of the leucine zipper disrupts both DNA binding by CBF1 and centromere function (*44*). CBF1 then contacts CDEI through basic residues of the bHLH, with His^227^, Glu^231^, and Arg^235^ of both CBF1 basic α helices recognizing bases of the near-palindromic CDEI motif, gtCAC[A/G]TG, in a sequence-specific manner (Fig. 3B). Notably, CBF1 binds to the CDEI motif in a manner that is identical to E-box (CACGTG) recognition by the metazoan heterodimeric bHLH Myc-Max transcription factor, using the same conserved amino acid triplet (Fig. 3B) (*45*, *46*). Mutation of either CBF1 residues mediating base-specific interactions, such as Glu^231^, or CBF1-binding nucleotides of CDEI disrupted CDEI-CBF1 interactions (*16*, *47*) and resulted in chromosome instability and hypersensitivity to microtubule poisons (*48*). Last, the CBF1 basic helices adopt an asymmetric dimer conformation to accommodate the CCAN structure, most apparent for subunit CBF1^A^ where the N-terminal basic α helix is unwound by nearly three turns to contact CENP-TW (Fig. 3A).

CCAN^Non-topo^ of the inner kinetochore complex engages the unwrapped DNA duplex (in this case, the 3′ end) of *C0N3*-CENP-A^Nuc^ through its CENP-LN DNA binding channel (Fig. 1, A to E). The binding mode of CCAN^Non-topo^, which has no associated CBF1, is the same as the CCAN assembled onto *W601*-CENP-A^Nuc^ determined previously (Fig. 1E and fig. S5B) (*34*). However, *C0N3*-CENP-A^Nuc^ is orientated differently in the CENP-LN channels of CCAN^Topo^ and CCAN^Non-topo^. For CCAN^Non-topo^, CENP-A^Nuc^ engages the Y-shaped opening of the complex end on, such that the histone octamer lies below the CENP-LN channel, sterically obstructing the raised conformation of CENP-HIK^Head^-TW in CCAN^Non-topo^ (Fig. 1E) (*34*). In contrast, for CCAN^Topo^, CENP-A^Nuc^ is rotated by ~150° about the unwrapped DNA duplex relative to CCAN^Topo^, bringing the histone octamer closer to CENP-LN and further unwrapping the DNA gyre to avoid CENP-A^Nuc^ clashing with CENP-LN. This allows space for the CENP-HIK^Head^-TW module of CCAN^Topo^ to adopt a raised conformation below the CENP-LN channel (Fig. 1D). Such a high degree of nucleosome unwrapping exposes basic residues of a H2A-H2B dimer responsible for binding DNA in a canonical H3 nucleosome (*49*). The removal of DNA-phosphate interactions from basic residues of H2A-H2B is partly compensated for by CCAN^Topo^ through acidic residues on the α3 helix of CENP-N (Fig. 3C).

CBF3^Core^ interacts with CCAN^Topo^ and CCAN^Non-topo^ mainly through their CENP-I subunits (Fig. 1, A and B). Specifically, the C terminus of CENP-I of CCAN^Non-topo^ forms contacts with the CEP3^A^ subunit of CBF3^Core^, which are unique to CCAN^Non-topo^ and not a feature of the CCAN^Topo^:CBF3 interaction (Fig. 1F). In the inner kinetochore complex, CBF3^Core^ adopts the same architecture as seen for free CBF3^Core^ (*50*–*53*), except that the Gal4-DNA binding domain of the CEP3^A^ subunit shifts position to interact with the essential CCG motif of CDEIII (Fig. 1C), similar to the complex of CBF3^Core^ with *CEN3*-CENP-A^Nuc^ (*36*). The cryo-EM density for the flexibly tethered CEP3^A^-Gal4 domain of CBF3^Core^ is diffuse, indicating that its interaction with the CDEIII motif as part of the inner kinetochore is weak (fig. S4F). Relative to the CENP-A^Nuc^:CBF3^Core^ cryo-EM structure solved recently (fig. S4C) (*36*), in our inner kinetochore complex, the remainder of CBF3^Core^ is displaced from the face of CENP-A^Nuc^ (fig. S4D). This allows space for the CENP-HIK^Head^-TW module of CCAN^Non-topo^ to adopt a position proximal to that face of CENP-A^Nuc^, which also prevents the scFv antibody from engaging the same nucleosome face in our reconstitutions.

The bridging of the two CCAN protomers by CBF3^Core^ (Fig. 1, A and B) suggests that CBF3^Core^ contributes to both the stability and organization of the assembled inner kinetochore complex. Consistent with this is our observation that a cryo-EM reconstruction of a complex comprising CBF1, CCAN^ΔC^, *C0N3*-CENP-A^Nuc^, and scFv (i.e., in the absence of CBF3^Core^) (fig. S1, K and L) comprised predominantly a monomeric CBF1:CCAN^Topo^:CENP-A^Nuc^ assembly (figs. S2F; S3D; S7, A and B; S8A, column b; and table S2). A monomeric CBF1:CCAN^Topo^:CENP-A^Nuc^ species is also observed at low occupancy, in the absence of scFv but with CENP-C bound (from the CBF1:CCAN:*C0N3*-CENP-A^Nuc^:CBF3^Core^ cryo-EM dataset), indicating that the topological entrapment of CENP-A^Nuc^ is not an artifact of scFv (fig. S7C). In the cryo-EM datasets of both for the inner kinetochore sample and when CBF3^Core^ was absent, a distinct pseudo-symmetric di-CCAN:CENP-A^Nuc^ species was observed at low occupancy, where both CCAN protomers adopt the CCAN^Non-topo^ configuration with no bridging CBF3^Core^ (figs. S8A, column c; S8B, column c; and S8D). For the inner kinetochore sample, however, which includes CBF3^Core^, the asymmetric di-CCAN configuration of the inner kinetochore reconstruction (Fig. 1) was the predominant species (fig. S8B, column d), consistent with the organizing role of CBF3^Core^.

### Model of the holo–inner kinetochore–CENP-A^Nuc^ complex with CENP-C and Ndc10^DBD^

To gain insights into the structure of the holo–inner kinetochore with CENP-C, we modeled CENP-C bound to IK*^C0N3^* based on the crystal structure of *S. cerevisiae* CENP-C^Cupin^ (*54*) and the cryo-EM structure of the CENP-C motif (residues 282 to 305) in complex with CENP-A^Nuc^ (PDB: 7ON1), combined with the AlphaFold2 prediction of *S. cerevisiae* CENP-C (*55*). Although scFv blocks the CENP-C binding site on one face of CENP-A^Nuc^, binding of two CENP-C subunits to their cognate binding sites on IK*^C0N3^* is compatible with the observed conformation and arrangement of CCAN and CBF3^Core^ subunits (fig. S4E). The Ndc10 component of CBF3 only weakly associates with CBF3^Core^ (*36*), and its inclusion in our inner kinetochore reconstitution resulted in heterogeneous complexes on cryo-EM grids. However, docking the Ndc10 DNA binding domain (Ndc10^DBD^; residues 27 to 538) onto IK*^C0N3^*, guided by the CBF3^Holo^ structure (*50*), indicated a position that generates complementary interfacial contacts with both CENP-I and CENP-L of CCAN^Non-topo^ (fig. S4E). This analysis suggests that our IK*^C0N3^* cryo-EM structure is likely representative of the holo–inner kinetochore complex with CENP-C and Ndc10.

### Disrupting inner kinetochore interfaces compromises chromosome segregation efficiency

We sought to assess the veracity of our inner kinetochore structure by testing the effects of mutants that disrupt either intersubunit or CCAN:CENP-A^Nuc^ interactions on the efficiency of minichromosome segregation and sensitivity to microtubule poisons in vivo. Mutation of CDEIII (*cdeIII^MT^*) and deletion of either CENP-N (*chl4*Δ) or CENP-I (c*tf3*Δ) severely compromised chromosome segregation efficiency (fig. S9, A to C). Mutations of basic residues of CENP-N that line the DNA binding channel (*chl4^MT1^*) also severely affected chromosome segregation efficiency (figs. S6B and S9B), consistent with previous results (*34*). Disruption of basic residues of CENP-I^Head^ (*ctf3^MT1^*) that participate in the topological DNA binding chamber (Fig. 2, B and C), significantly reduced chromosome segregation efficiency (figs. S6B and S9C). These results support our model that CBF1:CCAN engages a DNA duplex through the CENP-LN channel, augmented by contacts to CENP-I^Head^. Deletion of the CDEI motif (*cdeI^MT^*) resulted in a significant minichromosome loss (fig. S9A). To test the role of basic residues of the CBF1 bHLH motif, we replaced the wild-type *CBF1* gene with a *cbf1* mutant in which DNA binding residues were substituted with serines (*cbf1^MT2^*). Whereas wild-type CBF1 rescued the benomyl (a microtubule-destabilizing drug) sensitivity of a *cbf1*Δ strain, *cbf1^MT2^* did not (figs. S6B and S9D).

We also assessed the consequence of disrupting the CBF1:CENP-QU interface (fig. S6A, inset b). Introducing CBF1 mutations L283E and L287W (*cbf1^MT1^*) that contact CENP-Q^Ile192^ at the CBF1:CENP-QU interface generated sensitivity to benomyl, a phenotype identical to a CBF1 deletion (*cbf1*Δ) (figs. S6B and S9D). Mutating residues of the CENP-N α3 helix (*chl4^MT2^*) that interact with exposed basic residues of H2A-H2B (Fig. 3C) did not impair chromosome segregation efficiency significantly (figs. S6B and S9B). However, yeast strains with this CENP-N mutant showed increased sensitivity to benomyl (figs. S6B and S9E), suggesting that loss of these CENP-N:H2A-H2B interactions causes a mildly deleterious effect on kinetochore stability. Last, we tested the role of CBF3^Core^ in stabilizing the inner kinetochore complex. The essential CBF3 also targets CENP-A to centromeres (*56*), complicating its functional analysis in cells. Therefore, we focused on the CCAN interfaces and deleted the C-terminal 10 residues of CENP-I (*ctf3*^Δ*C10*^), which, in CCAN^Non-topo^, interact with the CEP3^A^ subunit of CBF3^Core^ (Fig. 1F). The *ctf3*^Δ*C10*^ mutant caused severe chromosome segregation defects (figs. S6B and S9, C and F), a result that supports our model of the inner kinetochore architecture in which CBF3^Core^ connects the two CCAN protomers through an interface with CENP-I^C-term^ of CCAN^Non-topo^ (Fig. 1). This is consistent with a recent in vivo study showing that CEP3 and CENP-A proteins colocalize to within 1.5 nm (indicative of being in the same complex) and are present at equal stoichiometry (*57*).

### The inner kinetochore reconstituted with native CEN3 DNA is comparable to *C0N3* DNA

We observed that the *C0N3* DNA used in the *C0N3*-CENP-A^Nuc^ reconstitution, in which the W601 sequence partially substitutes for CDEII of *CEN3*, is positioned identically to the *CEN3* sequence of *CEN3*-CENP-A^Nuc^ (fig. S4, A and B) (*36*). Specifically, the CDEI and CDEIII motifs in *C0N3*-CENP-A^Nuc^ have the same position relative to the nucleosome dyad as their counterparts in *CEN3*-CENP-A^Nuc^. *C0N3*-CENP-A^Nuc^ should therefore represent an effective and stable substitute of *CEN3*-CENP-A^Nuc^ for cryo-EM studies. Previous studies, however, had indicated that mutating CDEII results in mitotic delay and minichromosome segregation defects (*28*–*30*, *58*–*60*). We made similar observations in a chromosome segregation loss assay. A plasmid with a centromere based exactly on *C0N3* is substantially more prone to minichromosome mis-segregation than a native *CEN3*-based plasmid, albeit better than an acentromeric plasmid (*cen3*Δ) (fig. S9A). This indicated that *C0N3* does not fully recapitulate *CEN3* function in vivo, despite *C0N3*-CENP-A^Nuc^ having the same structure as the native *CEN3*-CENP-A^Nuc^ (*36*). Possible explanations for the impaired in vivo function of *C0N3* are the lower nucleosome occupancy associated with W601 sequences in vivo (*61*) and the recent finding that homopolymer AT runs are essential for efficient CENP-A deposition at yeast centromeres (*62*). As mentioned earlier, a native holo–inner kinetochore complex assembled using *CEN3* DNA and incorporating CENP-C without scFv, had a mass of 1.61 MDa, identical to the holo–inner kinetochore with *C0N3* DNA (fig. S1, D and G). We also prepared a *CEN3* inner kinetochore complex [CBF1:CCAN^ΔC^:*CEN3*-CENP-A^Nuc^:CBF3^Core^:scFv (IK*^CEN3^*)] for cryo-EM analysis, and as for the *C0N3* complex (IK*^C0N3^*), we replaced CENP-C with the scFv antibody (fig. S1J). The *CEN3* and *C0N3* inner kinetochore complexes also had similar compositions, eluting from the SEC column at identical volumes (fig. S1I). A cryo-EM dataset (fig. S8E) indicated that the *CEN3* inner kinetochore is not stable on cryo-EM grids, despite the scFv antibody, most likely because of the poor stability of *CEN3* nucleosomes, as also observed by others (*36*, *41*). While we observed 2D classes with low-particle occupancy for the pseudo-symmetric and asymmetric di-CCAN species (fig. S8C, columns c and d), the main species was a dimeric CCAN:DNA complex (fig. S8C, column a), devoid of intact CENP-A^Nuc^.

### A CENP-A^Nuc^-CENP-QU pathway is independent of CCAN

**Fig. 4.**
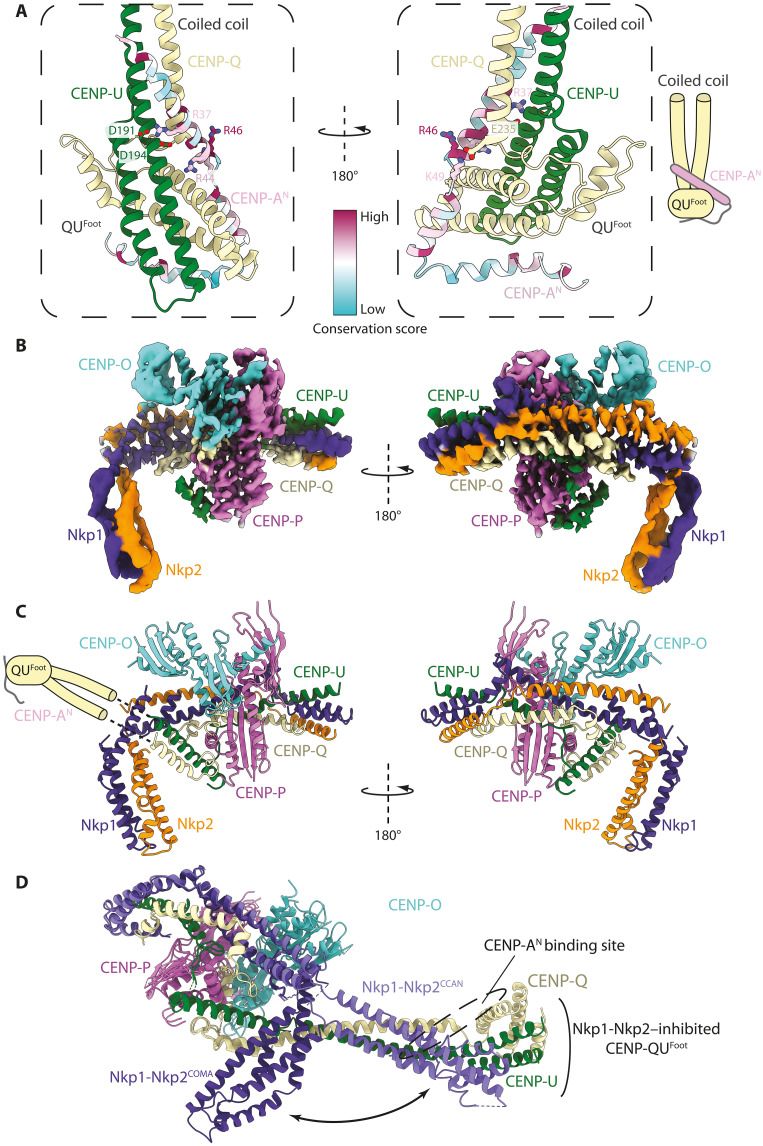
CENP-A^END^ interacts with CENP-QU autoinhibited by CCAN. (**A**) Two views of an AlphaFold2 model predicting how CENP-A^N^ interacts with CENP-QU. The major site of interaction involves CENP-A^END^ with CENP-QU^Foot^ and CENP-QU coiled coil. CENP-A^N^ is colored with a sequence conservation score. (**B**) Cryo-EM map of the CENP-A^N^:CENP-OPQU+ (COMA) complex. (**C**) Ribbons representation of the COMA complex. In this structure, the CENP-QU^Foot^ and adjacent coiled coil, including the CENP-A^END^ binding site of CENP-QU, are mobile and not visible in the cryo-EM map. These are shown schematically. (**D**) Structure of CENP-OPQU+ in the context of CCAN superimposed onto the COMA complex. This shows the conformational change of the N-terminal region of Nkp1-Nkp2 exposing the CENP-A^END^ binding site on CENP-QU (mobile) in the free CENP-OPQU+ complex (movie S2).

Previous studies identified an interaction between the essential N-terminal domain (END) of budding yeast CENP-A (CENP-A^END^; residues 28 to 60) (*37*, *38*) and the essential *S. cerevisiae* CCAN proteins CENP-Q and CENP-U (*39*, *40*). Reconstitution studies further showed that a CENP-QU module can transmit force from the outer kinetochore to CENP-A^Nuc^ (*63*). CENP-A^END^ bound CENP-QU with a dissociation constant (*K*_d_) ~ 0.7 μM measured using ITC, consistent with an earlier study (fig. S10, A to E) (*39*), and residues 1 to 82 of CENP-A (CENP-A^N^) formed a stable complex with CENP-QU as assessed by SEC (fig. S10G). We used AlphaFold2 (*64*) to predict the structure of a CENP-A^N^:CENP-QU complex (Fig. 4A and fig. S11, A to C). In the AlphaFold2 model, residues 21 to 80 of CENP-A form two α helices that embrace CENP-QU^Foot^ and contact the CENP-QU coiled coil (Fig. 4A), consistent with prior cross-linking mass spectrometry (CLMS) data (*39*, *40*). The longest of the two α helices (residues 21 to 61) contains the highly conserved CENP-A^END^ motif (Fig. 4A). Mutation of the basic CENP-A^N^ residues participating in the interaction interface (Fig. 4A) was previously shown to cause chromosome segregation defects (*37*), whereas the interacting residues of CENP-QU were shown to bind CENP-A (*39*, *40*). In further support of our AlphaFold2 model, SEC analysis showed that substituting Lys residues for residues for CENP-U^Glu191^, CENP-U^Glu194^, and CENP-Q^Asp235^ (CENP-QU^MT^), predicted to contact CENP-A^N^, disrupted the CENP-A^N^:CENP-QU complex in vitro (Fig. 4A and fig. S10, G and H). During the review of this paper, a preprint study reported the crystal structure of a trimeric complex of CENP-QU:CENP-A^END^ (*65*). This experimental structure validates our AlphaFold2 prediction (Fig. 4A).

Paradoxically, in the context of CCAN, the CENP-QU^Foot^ is sterically blocked by an N-terminal domain of the Nkp1-Nkp2 dimer (Fig. 4D and movie S2). Consistent with this, CENP-A^N^ did not bind either fully assembled CCAN or CENP-OPQU+ in the presence of CENP-LN (fig. S12, A and B), suggesting that CENP-LN promotes a conformational change in CENP-OPQU+, involving Nkp1-Nkp2, which blocks the CENP-A^END^ binding site on CENP-QU. To further assess this hypothesis, we determined a cryo-EM structure of the CENP-A^N^:CENP-OPQU+ (COMA) complex to 3.4 Å resolution (Fig. 4, B and C; fig. S11, D and E; table S2; and movie S2). In our structure, the N-terminal domain of Nkp1-Nkp2 is bent back, so only its middle region binds to CENP-QU (Fig. 4, C and D). The remodeled Nkp1-Nkp2 releases the coiled-coil domain of CENP-QU, rendering CENP-QU^Foot^ accessible to bind CENP-A^END^. Although the CENP-QU^Foot^ is not visible in the cryo-EM map due to conformational heterogeneity (Fig. 4B), this structure, together with the AlphaFold2 prediction, explains how CENP-A^END^ can bind CENP-QU in the context of Nkp1-Nkp2 and in the absence of CENP-LN.

**Fig. 5.**
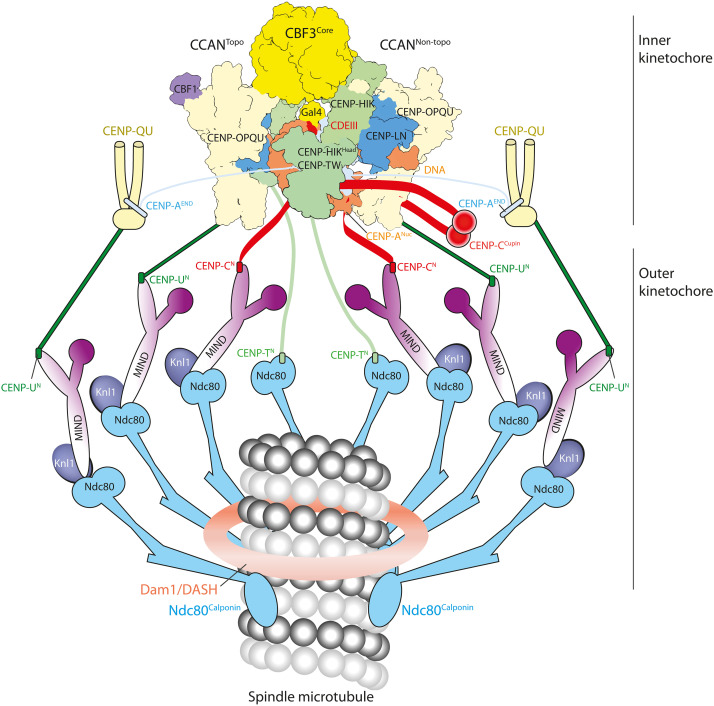
Schematic of the point centromere-kinetochore of *S. cerevisiae*. In this model, the holo–inner kinetochore complex (two CCAN protomers) together with two additional CENP-A^END^–binding CENP-QU modules form a total of eight Ndc80c connections to the spindle microtubule. CENP-QU:CENP-A^END^:MIND:Ndc80c modules represent components of the CENP-QU pathway described in Fig. 4. To which degree the CCAN and CENP-QU pathways overlap physically and temporally at the centromere remains to be determined. C^N^, U^N^, and T^N^ refer to the N-terminal motifs of CENP-C, CENP-U, and CENP-T, respectively, which bind MIND (C^N^ and U^N^) and Ndc80 (T^N^).

The CENP-A^END^–bound CENP-QU could represent a CCAN-independent axis of inner-outer kinetochore assembly. Consistent with this hypothesis, a CCAN:CENP-A^Nuc^ complex readily accommodated additional copies of CENP-QU, contingent on the N-terminal region of CENP-A (residues 1 to 129) (fig. S12, C and D) and consistent with preassembled CCAN not engaging CENP-A^N^ (fig. S12A). Collectively, our results suggest that budding yeast CENP-A^Nuc^ recruits two fully assembled CCAN protomers that associate with CBF1 and CBF3, as well as an additional two copies of CENP-QU (or CENP-OPQU+). Our data are consistent with observations that CENP-QU is present at supernumerary amounts at kinetochores in cells, recruiting additional copies of the MIND (Mtw1p including Nnf1p-Nsl1p-Dsn1p) and Ndc80 complexes to reinforce the load-bearing attachment (*66*). Last, *Cse4R37A* is a temperature-sensitive mutant (*67*) that, according to our structural model (Fig. 4A), would specifically weaken the CENP-A^END^:CENP-QU interaction. In agreement with this, replacing Arg^37^ with Ala reduced the affinity of a CENP-A^END^ peptide for CENP-QU 15-fold (fig. S10, B, D, and F). *Cse4R37A* is synthetically lethal when combined with either deletion or mutation of other nonessential CCAN genes, such as CENP-N (*67*), which are crucial for CCAN assembly (*34*). This further suggests that the CENP-A^N^:CENP-QU module and CCAN represent two distinct pathways to the outer kinetochore (Fig. 5).

## DISCUSSION

The topological entrapment of DNA by an enclosed chamber formed from CENP-LN:CENP-HIK^Head^-CENP-TW:CBF1 of CCAN^Topo^ in the yeast inner kinetochore complex reveals a mechanism of kinetochore attachment to centromeric chromatin that is evolutionarily conserved with how the human CCAN complex interacts with regional centromeres (*42*). For yeast CCAN, however, the topologically entrapped DNA duplex is the unwrapped DNA terminus of CENP-A^Nuc^, whereas human CCAN interacts with the linker DNA of the 171 bp α-satellite repeats of human centromeres (Fig. 2C). Substantial experimental studies previously demonstrated that native *S. cerevisiae* kinetochores consist of a single CENP-A^Nuc^ core (*8*, *11*, *12*, *43*), comprising two CENP-A histones (*5*, *37*, *68*–*70*), which is perfectly positioned on point centromeric sequences (*10*–*12*). In our structure, the CENP-A^Nuc^ core is flanked on both sides by a CCAN protomer that engage the unwrapped ends of the nucleosome in an asymmetric arrangement, bridged by CBF3^Core^. This assembly comprises two copies of all CCAN and histone subunits and of the Cep3 subunit of CBF3^Core^ (table S1), a stoichiometry that is in agreement with in vivo fluorescence microscopy–based assessments of the relative proportions of yeast inner kinetochore subunits, which are mainly found at approximately two to three copies per kinetochore (*57*, *66*, *69*–*72*). Specifically, CENP-A levels in budding yeast were estimated as two molecules per centromere in three in situ studies (*69*, *70*, *73*), a stoichiometry consistent with high-resolution chromatin immunoprecipitation and sequencing data (*8*, *11*, *12*) and our model of the inner kinetochore. An earlier in vivo study reported a mean of five CENP-A subunits associated with budding yeast centromeres (*72*). Recently, a single-molecule localization microscopy study estimated that four CENP-A molecules were present at individual budding yeast kinetochore complexes in situ (*57*). Because inhibiting protein synthesis, which specifically decreases non-centromeric CENP-A, reduced CENP-A protein copy number by 30 to 40%, this study would also be consistent with approximately one CENP-A^Nuc^ directly associated with the point centromere. The three additional CENP-A histones observed by Lawrimore and colleagues (*72*) were proposed to be randomly dispersed within pericentric DNA flanking the centromere, possibly by replacing H3 nucleosomes at non-*CEN* DNA (*41*, *74*); this does not exclude the one CENP-A nucleosome per centromere-kinetochore complex model. Additional CENP-A molecules may serve to maintain CENP-A nucleosomes at point centromeres (*75*), potentially also involving Ndc10 that directly recruits the CENP-A chaperone Scm3 involved in the deposition of CENP-A during S phase (*56*, *76*). Discrepancies between the three studies, finding two CENP-A histones at centromeres (*69*, *70*, *73*) and the two studies reporting four to five CENP-A molecules (*57*, *72*), might result from errors associated with methods used to calibrate CENP-A fluorescence signals with in vitro or exogenous green fluorescent protein (GFP) standards, the photophysical properties of GFP, the background correction, and the tendency of C-terminally GFP-tagged CENP-A to bind DNA nonspecifically (*69*, *70*). In vivo fluorescence studies also revealed higher levels of subunits of the COMA/CENP-OPQU+ subcomplex (*57*, *66*), a finding we attribute to the additional interactions between CENP-QU and CENP-A^END^, as discussed below. The cryo-EM structure of IK*^C0N3^* is consistent with the observed molecular mass of a holo–inner kinetochore complex reconstituted using native *CEN3* DNA and CENP-C (fig. S1G and table S1).

Both CCAN protomers of the inner kinetochore assembly engage the unwrapped DNA ends of CENP-A^Nuc^. While the CCAN^Topo^ protomer is positioned at CDEI, CCAN^Non-topo^ extends 30 bp 3′ of CDEIII, so that the total length of DNA embedded in the inner kinetochore complex is ~150 bp (Fig. 1, A and C). This matches almost exactly with the size and position of centromeric DNA protected from deoxyribonuclease I and micrococcal nuclease digestion of native budding yeast chromosomes, including protection of the region extending ~30 bp 3′ of CDEIII (*10*, *16*, *77*–*79*). Thus, our structure is in good agreement with prior characterization of native *S. cerevisiae* kinetochore-centromere complexes in vivo and in vitro and is further supported by the functional roles of specific residues tested in the in vivo assays reported here.

Numerous factors suggest that the inner kinetochore reconstituted using the near-native *C0N3* sequence in which W601 DNA substitutes for the AT-rich CDEII sequence is a good approximation of an inner kinetochore assembled on a native centromeric sequence. The positioning of the *CEN3* and *C0N3* sequences on CENP-A histone octamers is identical, *CEN3*-CENP-A^Nuc^ (*36*) and *C0N3*-CENP-A^Nuc^ have similar structures, including the non-centromeric CDEII region of *C0N3*-CENP-A^Nuc^, and as judged by SEC-MALS, the subunit composition and stoichiometry of holo–inner kinetochores reconstituted on *CEN3*-CENP-A^Nuc^ and *C0N3*-CENP-A^Nuc^ are indistinguishable from their expected masses. However, the defective segregation efficiency of minichromosomes harboring *C0N3* shows that the W601 sequence does not fully recapitulate the function of CDEII in vivo, results that are in agreement with previous studies investigating the function of CDEII (*28*–*30*, *58*–*60*). We suggest that, although native and *C0N3*-CENP-A^Nuc^ kinetochores likely have similar structures, differences between *CEN3* and *C0N3* sequences in vivo result from the homopolymer AT tracts of CDEII being more efficient for CENP-A deposition at native centromeres (*62*), a function possibly mediated by the AT-rich DNA binding domain of the CENP-A chaperone Scm3 (*76*). Last, the interaction of CENP-C with CDEII (*80*) might also contribute to optimal centromere function. The mechanism of how CENP-C and CDEII interact is not known and was not addressed in this study because of the use of the stabilizing scFv. However, our inner kinetochore structure readily accommodates a docked model of CENP-C onto CENP-A^Nuc^ (fig. S4E).

Cryo-EM reconstructions of both *S. cerevisiae* and human CCAN revealed that their underlying architectures are highly conserved, including the central DNA binding CENP-LN channel (*42*, *81*, *82*). However, this CENP-LN channel is notably wider in *S. cerevisiae* CCAN. As noted by others, on the basis of AlphaFold2 predictions of CENP-LN from a variety of species, a wider channel appears conserved in yeast using point centromeres, whereas the narrow CENP-LN channel is associated with organisms that evolved regional centromeres (*81*). Our structure of the *S. cerevisiae* inner kinetochore complex shows that only the wide CENP-LN channel is compatible with CCAN^Non-topo^, engaging CENP-A^Nuc^ in an end-on, nontopological configuration. Other differences between the architectures of point and regional kinetochores include the stoichiometry of two CCAN protomers to CENP-A^Nuc^ in budding yeast, in contrast to a single human CCAN protomer associated with either one or two α-satellite repeat CENP-A nucleosomes (*42*, *83*). Potentially, the single CENP-A^Nuc^ at point centromeres maximizes kinetochore-microtubule attachments by association with two CCAN protomers, as well as through the separate CENP-QU–outer kinetochore pathway described here. The reason why point centromere-kinetochore assemblies evolved two CCAN promoters asymmetrically organized on a central CENP-A^Nuc^ is not clear. It is possible that this arrangement of CCAN protomers is driven by the “asymmetric” organization of sequence motifs in the centromeric sequence, such as the CDEI element 5′ to the nucleosome dyad. CBF1 not only positions one of two CCAN modules at CDEI but also participates in topological entrapment through its acidic latch (Fig. 3A). It is plausible that only the CCAN module associated with CBF1 has the propensity to topologically entrap centromeric DNA. Moreover, in a symmetric arrangement of nontopological CCANs, only one protomer would bind CBF3^Core^, and this is perhaps less stable than the asymmetric CCAN dimer with CBF3^Core^. A symmetric arrangement of topological CCANs on the other hand would not require the wider CENP-LN channel; model docking suggests that, here, too, only one of the two CCAN^Topo^ protomers would bind CBF3^Core^. Last, because the interaction of CCAN^Topo^ with CENP-A^Nuc^ requires an additional 8 bp of unwrapped DNA (relative to CCAN^Non-topo^), a symmetric dimer with two CCAN^Topo^ protomers would involve increased DNA unwrapping and is therefore possibly less stable than other configurations.

Fourteen of the 16 budding yeast centromeres are between 117 and 119 bp in length, with *CEN4* being substantially shorter (111 bp) and *CEN12* the longest (120 bp) (*10*). Centromere length differences result from sequence variations in CDEII. As CDEII interacts with the dyad axis of the nucleosome, length variations in CDEII therefore predict that CDEI and CDEIII do not maintain the same position relative to the dyad axis in all 16 centromeres. Variations in CDEIII position relative to the dyad axis would require shifts in the Gal4-DNA binding domain of CBF3. We envision that this is readily accommodated by the flexible linker connecting the Gal4 domain to the main CEP3^A^ domain and that the DNA gyre of CENP-A^Nuc^ is unobstructed by CCAN protomers in the immediate vicinity of CDEIII^CCG^ (Fig. 1, A and C). Variations in CDEI position relative to the CENP-A^Nuc^ dyad axis, however, would involve a modest rotation of CBF1:CCAN^Topo^ relative to CENP-A^Nuc^, possibly accommodated by the flexible interfaces connecting CCAN, CENP-A^Nuc^, and CBF3 components, as observed in our inner kinetochore cryo-EM reconstruction (fig. S3, A and B).

By providing insight into the mechanism of CENP-A^END^ interactions with CENP-QU (Fig. 4), we characterize two independent CENP-A^Nuc^ pathways to the outer kinetochore: (i) a direct link from CENP-A^END^ through CENP-U to MIND, independent of CCAN, and (ii) a CCAN:CENP-A^Nuc^ pathway linking CENP-U to MIND and CENP-T to Ndc80c (Fig. 5). Our structure now provides a compelling estimate for the probable stoichiometry of the inner and outer kinetochore. Up to six attachment points for the MIND and Ndc80c outer kinetochore modules are presented by CCAN^Topo^ and CCAN^Non-topo^ (Fig. 5): two CENP-C molecules (binding a MIND:Ndc80c module each; fig. S4E), two CENP-T molecules (binding an Ndc80c each), and two CENP-QU modules (binding a MIND:Ndc80c module each) (*84*). Taking into account the CENP-A^Nuc^-CENP-QU pathway described above, an additional two MIND:Ndc80c module connection points are generated (Fig. 5). Fluorescence microscopy studies estimate the number of MIND and Ndc80c components per kinetochore at ∼6 to 7 and ∼8 to 10, respectively (*57*, *66*, *71*).

In conclusion, our structure of the budding yeast inner kinetochore reconstituted onto a CENP-A nucleosome provides a foundation for understanding the higher-order centromere-kinetochore assembly and has implications for how a single CENP-A^Nuc^ coordinates assembly of multiple MIND and Ndc80 complexes. Our study answers long-standing questions of how the defined sequence elements of point centromeres engage sequence-specific DNA binding complexes to organize the load-bearing attachments of the inner kinetochore.

## MATERIALS AND METHODS

### Peptide synthesis

CENP-A^END^ peptides were synthesized by Cambridge Research Biochemicals at 95% purity. All three peptides contained N-terminal acetylation and C-terminal amidation and were at 95% purity (W) = peptide with an additional C-terminal tryptophan was used to confirm peptide concentrations in stoichiometry studies. CENP-A^END-1^: DASINDRALSLLQRTRATDAW, residues 32 to 48; CENP-A^END-2^: AGDQQSINDRALSLLQRTRATKNW, residues 28 to 50; CENP-A^END-2-R37A^: AGDQQSIND**A**ALSLLQRTRATKNW, residues 28 to 50; and CENP-A^END-3^: AGDQQSINDRALSLLQRTRATKNLFPRREERRRW, residues 28 to 60.

### Isothermal titration calorimetry

ITC was performed using an Auto-iTC200 instrument (Malvern Instruments, Malvern, UK) at 20°C. CENP-A^END-1^, CENP-A^END-2^, and CENP-A^END-3^ have *K*_d_ of 11.5, 1.0, and 0.72 μM, respectively. Peptide concentrations are as follows: (i) 2.18 mM, (ii) 0.87 mM, (iii) 1.18 mM, and (iv) 1.0 mM. Buffer contains the following: 20 mM Hepes (pH 7.5), 100 mM NaCl, and 1 mM tris(2-carboxyethyl)phosphine (TCEP). For each titration run, 370 μl of CENP-QU (between 66 and 180 μM) was used to load the calorimeter cell. The CENP-A^END^ peptides at 0.87 to 2.18 mM were titrated into the cell consisting of one 0.5 μl injection followed by 19 injections of 2 μl each. After discarding the initial injection, the changes in the heat released were integrated over the entire titration and fitted to a single-site binding model using the MicroCal PEAQ-ITC Analysis Software 1.0.0.1258 (Malvern Instruments). Titrations were performed in triplicate.

### Cloning

All genes and proteins used in this study are of *S. cerevisiae* origin. Expression constructs and systems for assembly of the CENP-OPQU+, CENP-HIK-TW and CENP-LN subcomplexes, CENP-C, and the CENP-A octamer were described in (*34*) (table S1). For the CENP-A^∆N^ octamer preparation, the expression cassette of CENP-A^130–229^ was combined with *H2A*, *H2B*, and *H4* expression cassettes in a single pET28 plasmid. The CBF3^Holo^ complex was prepared as described in (*50*). The CBF3^Core^ complex (*Cep3*, *Ctf13*, and *Skp1*) was cloned into pU2, and Cbf1 was cloned into pU1 (*85*). Cep3 and Cbf1 were cloned with C-terminal tobacco etch virus (TEV)–cleavable double StrepII tags as described in (*85*). For the CENP-ΔQU complex, the coding sequences of CENP-Q^1–294^ (CENP-ΔQ) and CENP-U^30–266^ (CENP-ΔU) were cloned into pET28 plasmids, with a TEV-cleavable double StrepII tag on the CENP-U C terminus. The two cassettes were further combined into a single pET28 plasmid. For the CENP-A^N^ protein, the coding region of CENP-A^1–82^ was cloned into a pAcycDuet plasmid with a N-terminal 3C protease cleavable His_6_ tag. For the single-chain antibody, the scFv coding sequence (*36*) was synthesized (Thermo Fisher Scientific) and subcloned into pET28A.

### Protein and complex preparation

CCAN subcomplexes (CENP-C, CENP-LN, CENP-OPQU+, and CENP-HIK-TW) were expressed in the insect cell–baculovirus system, CENP-A and CENP-A^∆N^ octamers in *Escherichia coli*, and purified as described in (*34*). CBF1, CBF3^Core^, CBF3^Holo^, and Ndc10 were expressed individually in High-5 insect cells. Cells were harvested 48 hours after infection. The cleared lysate was loaded onto an affinity column [either Strep-Tactin column (QIAGEN) or HisTrap HP column (QIAGEN)] for purification of expressed proteins and subcomplexes. The tags were cleaved by a 16-hour incubation with TEV protease at 4°C. The protein complexes were then purified by Resource Q anion exchange, and further purified by SEC in a buffer of 20 mM Hepes (pH 8.0), 300 mM NaCl, and 0.5 mM TCEP.

CENP-ΔQU complex expression was performed at 20°C for 16 hours with 0.36 mM isopropyl-β-d-thiogalactopyranoside in *E. coli* strain B834 with codon plus Rare2. The complex was purified by a combination of Strep-Tactin (QIAGEN) in a buffer of 50 mM tris-HCl (pH 8.0), 250 mM NaCl, 1 mM EDTA, and 1 mM dithiothreitol (DTT); followed by cation exchange chromatography Resource S (Cytiva) with buffer of 20 mM tris-HCl (pH 7.5), 75 mM NaCl, 1 mM EDTA, and 1 mM DTT (gradient elution with buffer containing 1 M NaCl); and Superdex 200 SEC (Cytiva) with buffer of 10 mM tris-HCl (pH 7.5), 150 mM NaCl, 1 mM DTT, and 1 mM EDTA. The complex was concentrated to 8 mg/ml and stored at −80°C.

CENP-A^N^ was expressed as for CENP-QU. The protein was purified by nickel-nitrilotriacetic acid (Ni-NTA) with buffer of 50 mM tris-HCl (pH 8.0) and 250 mM NaCl, eluted with the buffer containing 300 mM imidazole. CENP-A^N^ was further separated by Superdex 75 SEC (Cytiva) with buffer of 10 mM tris-HCl (pH 7.5), 150 mM NaCl, 1 mM DTT, and 1 mM EDTA. The protein was concentrated to 2 mg/ml and stored at −80°C.

The scFv was prepared using a protocol adapted from (*36*). The inclusion bodies that contain scFv were prepared from overexpressing scFv from a pET28A plasmid in *E. coli* B834^rare2^ cells. The inclusion bodies were solubilized with a denaturation buffer of 100 mM tris-HCl (pH 8.0), 6 M guanidine buffer, and 2 mM EDTA and then spun down. The supernatant was adjusted to a protein concentration to 10 mg/ml. 1,4-Dithioerythritol powder was added to a final concentration of 10 mg/ml and shaken at 20°C for 16 hours. While stirring, 10 ml of the supernatant was quickly added to 1 liter of prechilled (10°C) refolding buffer and stirred for 3 min. The refolding buffer contains freshly added oxidized glutathione powder (551 mg/liter) in 100 mM tris-HCl (pH 9.5), 1 mM EDTA, and 0.5 M arginine (pH 9.5). The refolding solution was incubated at 10°C for 48 hours without stirring. One liter of refolding solution was then dialyzed against 5 liters of prechilled (4°C) dialysis buffer of 20 mM tris-HCl (pH 7.4) with 34 g of urea (added before dialysis) with a 6 to 8 kDa cutoff dialysis tubing for 16 hours at 4°C. This dialysis step was then repeated using fresh buffer. The refolding solution was filtered through a 0.22 μM filter unit then mixed with 4 ml of pre-equilibrated SP Sepharose Fast Flow resin in SP-binding buffer of 20 mM tris-HCl (pH 7.4) for 1 hour at 4°C. The resin was collected with an Econo column and washed with SP-binding buffer, and scFv was eluted using 360 mM NaCl in the SP-binding buffer. The scFv was further purified on a Superdex S75 size exclusion column, concentrated to 1 mg/ml, and stored at −80°C in a buffer of 20 mM tris-HCl (pH 7.4), 150 mM NaCl, and 1 mM EDTA.

### DNA generation

The 153 bp *C0N3* DNA fragment was prepared by the primer extension method. Oligos of C0N3 (forward) ATAAGTCACA TGGTGCCGAG GCCGCTCAAT TGGTCGTAGA CAGCTCTAGC ACCGCTTAAA CGCACGTA CG CGCTGTCCCC CGCG TTTTAA and C0N3 (reverse) TTCAATGAAA TATATATTTC TTACTATTTC TTTTTTAACT TTCGGAAATC AAATACACTA ATATTAAAAC GCGGGGGACA GCGCGTACGT were synthesized by Sigma-Aldrich. After mixing the oligos in a 1× polymerase chain reaction mixture, the fragment was produced with one-step extension at 68°C for 1 min. The final product of the 153 bp *C0N3* ATAAGTCACA TGGTGCCGAG GCCGCTCAAT TGGTCGTAGA CAGCTCTAGC ACCGCTTAAA CGCACGTACG CGCTGTCCCC CGCGTTTTAA TATTAGTGTA TTTGATTTCC GAAAGTTAAA AAAGAAATAG TAAGAAATAT ATATTTCATT GAA fragment was purified using 1 ml of Resource Q anion exchange chromatography and stored in a buffer of 2 M NaCl, 10 mM tris-HCl (pH 7.5), 1 mM EDTA, and 2 mM DTT at −20°C.

For the 153 bp *CEN3* DNA fragment, three copies of a fragment (ATAAGTCACA TGATGATATT TGATTTTATT ATATTTTTAA AAAAAGTAAA AAATAAAAAG TAGTTTATTT TTAAAAAATA AAATTTAAAA TATTAGTGTA TTTGATTTCC GAAAGTTAAA AAAGAAATAG TAAGAAATAT ATATTTCATT GAA) flanked by Eco RV site were cloned into pUC19. The plasmid was isolated by using the Plasmid Giga Kit (QIAGEN). The *CEN3* fragment was purified with a 1 ml Resource Q anion exchange chromatography column (Cytiva) after digestion with Eco RV-HF (New England Biolabs) for 16 hours. The purified DNA was precipitated, dissolved, buffer-exchanged, and stored in a buffer of 2 M NaCl, 10 mM tris-HCl (pH 7.5), 1 mM EDTA, and 2 mM DTT at −20°C.

### CENP-A nucleosome and derivative preparation

CENP-A and CENP-A^∆N^ nucleosomes were prepared by wrapping the prepared octamers with *C0N3* DNA or *CEN3* DNA by gradient dialysis. Either CENP-A or CENP-A^ΔN^ octamers were mixed with either *C0N3* DNA or *CEN3* DNA all at 7.8 μM. The mixture was dialyzed from 2 M NaCl to 100 mM NaCl in 10 mM tris-HCl (pH 7.4), 1 mM EDTA, and 2 mM DTT buffer for at least 16 hours at 20°C. The mixture was further dialyzed in a buffer of 10 mM tris-HCl (pH 7.4), 1 mM EDTA, and 2 mM DTT for 4 hours. For the *CEN3*-CENP-A nucleosome, the final dialysis step was performed at 65°C for 4 hours and then spun down for 1 min to remove aggregates at 4°C. The wrapped nucleosomes were assessed on native agarose gels and stored at 4°C.

### Assembly of IK*^C0N3^* and IK*^CEN3^* complexes

CENP-A nucleosome was mixed with CCAN subcomplexes: CENP-LN, CENP-OPQU+, CENP-HIK-TW, CBF1, and CBF3^Core^ at 2 μM concentration. The mixture was dialyzed in a buffer of 20 mM Hepes (pH 8.0) and 80 mM NaCl for at least 5 hours to remove DTT or TCEP. scFv (4 μM) was then added, and the sample was dialyzed against a buffer of 20 mM Hepes (pH 8.0) and 50 mM NaCl for 14 hours at 4°C. The complex was then concentrated to 3 mg/ml. To stabilize the complexes, 3 mM BS3 was used to cross-link the complex for 30 min on ice. The reaction was quenched by 50 mM tris-HCl (pH 8.0) and incubated on ice for 20 min. The mixture was applied to an Agilent 1000 Å column to remove excess CCAN subcomplexes before preparing cryo-EM grids. Uncross-linked complex was also loaded on to an Agilent 1000 Å column to access the quality of the assembled complex. The same procedure was used for assembly of CBF1:CCAN^ΔC^:*C0N3*-CENP-A^Nuc^:scFv, but without CBF3^Core^.

### Assembly of holo–inner kinetochore complexes: CBF1:CCAN:*C0N3*-CENP-A^Nuc^:CBF3^Core^ and CBF1:CCAN:*CEN3*-CENP-A^Nuc^:CBF3^Core^

As for IK*^C0N3^* and IK*^CEN3^* except that CENP-C was included with CCAN subcomplexes when mixed with CENP-A^Nuc^, scFv was omitted.

### Testing supernumerary CENP-QU binding to CCAN:CENP-A nucleosome complexes mediated through CENP-A^N^

CENP-A or CENP-A^∆N^ nucleosomes were wrapped with *C0N3* DNA. The nucleosomes were then mixed with CCAN components (CENP-C, CENP-LN, CENP-OPQU+, and CENP-HIK-TW) to form CCAN:*C0N3*-CENP-A and *C0N3* CENP-A^∆N^ nucleosome complexes. CENP-ΔQU was mixed with either of the two complexes at 2 μM in a buffer of 20 mM Hepes (pH 8.0), 80 mM NaCl, and 0.5 mM TCEP for 2 hours. The mixtures were then loaded onto an Agilent 1000 Å column. The peak fractions were visualized by 4 to 12% on an SDS–polyacrylamide gel electrophoresis (SDS-PAGE) gel stained with Instant Blue Coomassie.

### CENP-OPQU+:CENP-A^N^ sample preparation for cryo-EM

To generate CENP-OPQU+:CENP-A^N^ complexes, 10 μM of CENP-OPQU+ was incubated with 10 μM CENP-A^N^ in buffer of 20 mM Hepes (pH 8.0), 80 mM NaCl, and 0.5 mM TCEP on ice for 1 hour and then loaded onto an Agilent 1000 Å column. The eluted samples were visualized by SDS-PAGE stained with Instant Blue Coomassie. To prepare cryo-EM grids, the CENP-OPQU+:CENP-A^N^ complex was cross-linked by incubation in 3 mM BS3 on ice for 30 min, followed by quenching with 50 mM tris-HCl (pH 8.0) on ice for 20 min.

### Assessment of CENP-A^N^ binding to CENP-OPQU+ in the presence of CENP-LN

To test the effect of CENP-LN on the CENP-OPQU+:CENP-A^N^ complex, CENP-A^N^, CENP-OPQU+, and CENP-LN were mixed at 4 μM each in a buffer of 20 mM Hepes (pH 8.0), 80 mM NaCl, and 0.5 mM TCEP and loaded onto a Superose 6 size exclusion column.

### Testing binding of CENP-A^N^ to CCAN

To test the binding of CENP-A^N^ to CCAN, CENP-A^N^ (2.5 μM) and CCAN components (CENP-C, CENP-LN, CENP-OPQU+, and CENP-HIK-TW) (2.0 μM) were mixed in a buffer of 20 mM Hepes (pH 8.0), 80 mM NaCl, and 0.5 mM TCEP and loaded onto an Agilent 1000 size exclusion column.

### Size exclusion chromatography–multiangle light scattering

SEC-MALS was performed using an Agilent 1200 series LC system with an online Dawn Helios ii system (Wyatt) equipped with a QELS+ module (Wyatt) and an Optilab rEX differential refractive index detector (Wyatt). CENP-A nucleosome (either *CEN3*-CENP-A^Nuc^ or *C0N3*-CENP-A^Nuc^) and all the CCAN subcomplexes—CENP-C, CENP-LN, CENP-OPQU^+^, and CENP-HIK-TW—together with CBF1 and CBF3^Core^ complexes were mixed at 2 μM concentration to generate the complete inner kinetochore assembly. The mixture was dialyzed in a buffer of 20 mM Hepes (pH 8.0), 80 mM NaCl, and 0.5 mM TCEP for at least 5 hours. The inner kinetochore sample was then cross-linked with 3 mM BS3 for 30 min. The cross-linked sample was purified on an Agilent 1000 Å column. The peak fractions were concentrated, and 100 μl was injected onto an Agilent Bio SEC-5 column gel filtration column pre-equilibrated in 10 mM Hepes (pH 8.0), 80 mM NaCl, 1 mM EDTA, and 0.5 mM TCEP. The light scattering and protein concentration at each point across the peaks in the chromatograph were used to determine the absolute molecular mass from the intercept of the Debye plot using Zimm’s model as implemented in the ASTRA v7.3.0.11 software (Wyatt Technologies). To determine interdetector delay volumes, band-broadening constants, and detector intensity normalization constants for the instrument, thyroglobulin was used as a standard before sample measurement. Data were plotted with the program PRISM v8.2.0 (GraphPad Software Inc.).

### Minichromosomal stability assay

The minichromosomal stability assay was based on a method described previously (*50*). A fragment of *ARS1-TRP1-CEN3* was cloned into the *pUC18* plasmid to generate a *CEN3* minichromosome (wild type: *CEN3*). On the basis of *CEN3*, *cdeIII*^MT^ was generated by exchanging CCG to AGC. *cdeI*^MT^ was created by exchanging its GTCACATG to AATTGGCT. The *C0N3* minichromosome was generated by exchanging its *CEN3* with *C0N3*. The sequence of *CEN3* was removed from *CEN3* for the *cen3*Δ minichromosome control. This set of minichromosomes was transformed into BJ2168 and selected with Sc-TRP (yeast synthetic medium drop out tryptophan) plates. A single colony from each was cultured in nonselective yeast extract, peptone, and dextrose (YPD) medium for 12 hours. The cultures were diluted and spread onto YPD plates and grown for 3 days to obtain single colonies. The colonies were then plated onto Sc-TRP plates and incubated for 3 days at 30°C, and the selected colonies were counted to determine the percentage of minichromosome retained.

The BJ2168*^CEN3^* strain was used for deletion of the *CBF1*, *CTF3*, and *CHL4* genes by replacing their respective coding sequences with the KanMX6 gene to create the BJ2168^*CEN3,cbf1*∆^, BJ2168*^CEN3,ctf3∆^*, and BJ216*^CEN3,chl4∆^* strains by selection on G418 plates. The knockout strains were confirmed by sequencing.

*CBF1*, *CTF3* (*CENP-I*), and *CHL4* (*CENP-N*) genes were cloned into the pYes2 plasmid along with their native promoters and the *URA3* selection marker. *cbf1*^*MT1*(L283E,L287W)^, *cbf1*^*MT2*(K224S,K228S,R234S,R235S,K256S)^, *ctf3*^*MT1*(R215S,K216S,K219S,R222S,K225S)^, *ctf3*^ΔC10(F719S,∆724–733)^, *chl4*^MT1(K22S,K26S,R67S,K100S,K103S,K105S,R198S,K217S,K245S,K249S,K384S,K401S,K403S)^, and *chl4*^MT2(D48R,D50R,E56R,E63R)^ mutants were created from their wild-type constructs. These plasmids were transformed into the appropriate BJ2168*^CEN3^* knockout strain to create BJ2168*^CEN3,cbf1∆,CBF1^*, BJ2168*^CEN3,cbf1∆,cbf1-MT1^*, BJ2168*^CEN3,cbf1∆,cbf1-MT2^*, BJ2168*^CEN3,ctf3∆,CTF3^*, BJ2168*^CEN3,ctf3∆,ctf3MT1^*, BJ2168*^CEN3,ctf3∆,ctf3-MT2^*, BJ2168*^CEN3,chl4∆,CHL4^*, BJ2168^C*EN3,chl4,chl4-MT1*^, and BJ2168*^CEN3,chl4∆,chl4-MT2^* strains. The empty pYes2 plasmid was transformed into the BJ2168*^CEN3,cbf1∆^*, BJ2168*^CEN3,ctf3∆^*, and BJ2168*^CEN3,chl4∆^* strains as a control. Transformed yeast strains were selected on Sc-TRP-URA (Sc-TRP and uracil) plates.

Single colonies (20 per experiment; *n* = 20) of the above BJ2168 strains were cultured in Sc-URA (nonselective for minichromosome) for 16 hours. The cultures were diluted and plated onto Sc-URA plates and incubated for 3 to 6 days at 30°C to obtain single colonies. These colonies were restoked onto Sc-TRP-URA plates, incubated for 3 to 6 days at 30°C. Selected colonies were counted to determine the percentage of minichromosome retained. The experiments were performed independently at least eight times. Data were analyzed using Prism 9 (version 9.5.1; GraphPad), *n* = 20. Data in all groups (wild type and associated mutants) in each of the three datasets were included in a family-wise comparison analysis using ordinary one-way analysis of variance (ANOVA) Tukey’s multiple comparisons test (10 comparisons per family). The corresponding adjusted *P* values are indicated. The mean is indicated for each group. Error bars show SEM. Data are presented as a scatterdot plot.

### Benomyl sensitivity assay

The method was based on published studies (*86*). Freshly grown single colonies on Sc-URA plates were suspended in water adjusted to 1 × 10^6^ cell/ml. The cells (in a one-fifth dilution series) were grown on YPD and benomyl (25 μg/ml). After incubation at 25°C for 6 days, the plates were photorecorded.

### Immunoprecipitation and Western blotting for detecting the expression of CBF1, CENP-N, and CENP-I and their respective mutants

The yeast strains were cultured in synthetic complete dropout URA and TRP media (empty pYes2-URA3 vector control) and collected at an OD_600_ (optical density at 600 nm) of approximately 0.8. Pelleted cells were lysed in buffer [50 mM tris-HCl (pH 8.0), 300 mM NaCl, 1 mM EDTA, and 1 mM DTT], and the cleared lysate was loaded onto a 1 ml Strep-Tactin column. Fractions were eluted with 5 mM desthiobiotin and analyzed by SDS-PAGE. Western blotting was performed with a Strep-tag antibody (MCA2489P, Bio-Rad) that detected the C-terminal double StrepII tag on CBF1, CENP-N, and CENP-I. Total protein was analyzed by Coomassie blue staining for loading controls (normalized loading).

### Cryo-EM grid preparation

For all complexes, 0.05% (w/v) β-OG (*n*-octyl-β-d-glucopyranoside) was added to the sample immediately before plunge freezing. Three microliters of sample was applied to r2/2 Quantifoil mesh 300 grids, and after 20 s of incubation, the excess sample was blotted away and grids were plunge-frozen in liquid ethane [blot force of −10, blot time of 2 s, 4°C, 100% humidity, Vitrobot Mark IV (Thermo Fisher Scientific)]. The grids were screened on a 200 kV Glacios (Thermo Fisher Scientific), and movies were recorded on a 300 kV Titan Krios (Thermo Fisher Scientific) with a Falcon IV (Thermo Fisher Scientific) or K3 (Gatan) direct electron detector [Electron Bio-imaging Centre (eBIC) and Medical Research Council Laboratory of Molecular Biology (MRC-LMB)]. Data collection parameters and metrics are listed in table S2.

### Cryo-EM analysis, model building, and refinement

For the CCAN-containing complexes, all processing steps were carried out in RELION 4.0 (*87*). Motion correction was carried out with RELION 4.0, and contrast transfer function (CTF) estimation with CTFFIND4 (*88*). Particles were picked with Topaz (*89*). After extensive 2D classification (fig. S3) and 3D classification, 43,467 particles were used for 3D refinement of CBF1:CCAN:*C0N3*-DNA, 100,311 particles for 3D refinement of CBF1:CCAN^ΔC^:*C0N3*-CENP-A^Nuc^:scFv, and 108,672 particles for 3D refinement of IK*^C0N3^* (table S2).

For the IK*^C0N3^* dataset, consensus refinements were limited to 5.6 Å resolution, locally ranging from 4.2 to 15 Å, due to conformational heterogeneity (fig. S3A). Multibody refinement with four rigid bodies was set up to increase the resolution (body 1: CCAN^Topo^, body 2: CCAN^Non-topo^-^ΔCENP-I(Body)^, body 3: CBF3^Core^ + CENP-I^Body^, and body 4: CENP-A^Nuc^). All bodies refined to 3.7 to 3.8 Å resolution (fig. S3B) with clear side chain density for most regions within each body. These individual multibody maps were combined to generate a composite cryo-EM density map. For the CBF1:CCAN:*C0N3*-DNA dataset, masked 3D classification revealed a subset of 43,467 particles with a well-resolved density for CENP-HIK^Head^-TW (fig. S3C), which resulted in 3.4 Å resolution reconstruction after 3D refinement, Bayesian polishing, and per-particle CTF refinement.

For the CBF1:CCAN^ΔC^:*C0N3*-CENP-A^Nuc^:scFv dataset, consensus refinements after Bayesian polishing and per-particle CTF refinement resulted in a well-resolved density for CBF1:CCAN but diffuse density for the CENP-QU^Foot^ and CENP-A^Nuc^ because of conformational heterogeneity. To improve the reconstructions of our conformationally heterogeneous particle sets, we applied a variational autoencoder that is similar to the Gaussian mixture approach proposed in (*90*), where conformational variability in the data is mapped to a small latent space. For a given latent coordinate, which describes the conformation of an individual particle in the dataset, the decoder predicts a 3D deformation that acts on a collection of Gaussian-shaped pseudo-atoms that approximates the reconstructed density. Unique to our approach, once the 3D deformations were estimated for the entire dataset, we trained a second neural network that approximates the inverse of those transformations. We then use a real-space weighted-back projection algorithm, where the original particles are back-projected along lines deformed by the inverse transformations, to obtain an improved reconstruction (details to be published elsewhere by S.H.W.S. and J.S.) (fig. S3D).

For the CENP-OPQU+:CENP-A^N^ complex, micrograph movie frames were aligned with MotionCor2, and CTF estimation was performed by CTFFIND4. Particle picking was performed using a general model in Topaz (*89*). Extracted particles were initially subjected to 2D classification in cryoSPARC v3.4. Ab initio maps were then refined using homogeneous refinement, and the resulting map was further refined using nonuniform refinement. Particles that generated the best-resolved volume were used for training a new Topaz model to improve particle picking. Newly picked particles were used as input in two rounds of heterogeneous refinement against one true map obtained from nonuniform refinement and five noisy, decoy maps and subsequent 2D classification. The final consensus map at 3.4 Å resolution was generated through nonuniform refinement, and a small amount of anisotropy was observed.

A CBF1 monomer was modeled with AlphaFold2 (*64*) and docked into the cryo-EM map as a homodimeric bHLH with Coot (*91*). Existing CCAN:CENP-A^Nuc^ (PDB ID: 6QLD) (*34*) and CBF3^Core^ (PDB ID: 6GYP) (*50*) structures were docked into the respective cryo-EM maps and adapted to fit the density with Coot. All structures were refined manually in Coot and with Phenix (table S2) (*92*). Figures were generated using ChimeraX (*93*).

For the CENP-OPQU+:CENP-A^N^ complex, CENP-OPQU+ from the previously determined apo-CCAN structure (PDB ID: 6QLF) (*34*) was rigid-body fitted into the CENP-OPQU+ map using Chimera. CENP-OPQU+ was then manually modified using Coot, repositioning the Nkp1-Nkp2 domain and removing flexible loops not visible in the cryo-EM density maps. The final model was refined in Phenix (*92*) using default settings and model restraints from the apo-CCAN structure (PDB ID: 6QLF) (*34*).

### Negative-stain EM

Negative-stain EM grids of the non–cross-linked IK*^C0N3^* sample were prepared using 0.1 mg/ml of the complex stained with 2% uranyl acetate. A total of 776 images were collected on an F20 electron microscope (Thermo Fisher Scientific) equipped with a Falcon III detector. Data were processed with RELION 4.0.

### AlphaFold2 predictions

AlphaFold-Multimer (*64*, *94*) was run to predict models for structures of the CENP-A^N^:CENP-QU and CENP-A^N^:CENP-QU:Nkp1-Nkp2 complexes. Full-length CENP-Q, CENP-U, Nkp1, Nkp2, and residues of 1 to 120 of CENP-A were used in the prediction.

*Note added in proof*: After the manuscript was accepted for publication, the authors requested the following two references that refer to two programs used in the cryo-EM processing be added: 

A. Punjani, J. L. Rubinstein, D. J. Fleet, M. A. Brubaker, cryoSPARC: algorithms for rapid unsupervised cryo-EM structure determination. *Nat. Methods* **14**, 290–296 (2017). https://doi.org/10.1038/nmeth.4169. 

S. Q. Zheng, E. Palovcak, J.-P. Armache, K. A. Verba, Y. Cheng, D. A. Agard, MotionCor2: anisotropic correction of beam-induced motion for improved cryo-electron microscopy. *Nat. Methods* **14**, 331–332 (2017). https://doi.org/10.1038/nmeth.4193.

## Supplementary Materials

### This PDF file includes:

Figs. S1 to S12 Tables S1 and S2 Legends for movies S1 and S2 References
