# Supplementary material for: Cryo-EM structure of the complete inner kinetochore of the budding yeast point centromere
Source: Sci Adv. 2023 Jul 28;9(30):eadg7480. doi: 10.1126/sciadv.adg7480 (PMC10381965; doi:10.1126/sciadv.adg7480)
Supplement: Supplementary file 1 — Figs. S1 to S12 Tables S1 and S2 Legends for movies S1 and S2 References [file sciadv.adg7480_sm.pdf]

Science Advances
AAAS

Supplementary Materials for

Cryo-EM structure of the complete inner kinetochore of the budding yeast point centromere

Tom Dendooven *et al.*

Corresponding author: David Barford, dbarford@mrc-lmb.cam.ac.uk

Sci. Adv. **9**, eadg7480 (2023)
DOI: 10.1126/sciadv.adg7480

The PDF file includes:

Figs. S1 to S12
Tables S1 and S2
Legends for movies S1 and S2
References

Other Supplementary Material for this manuscript includes the following:

Movies S1 and S2

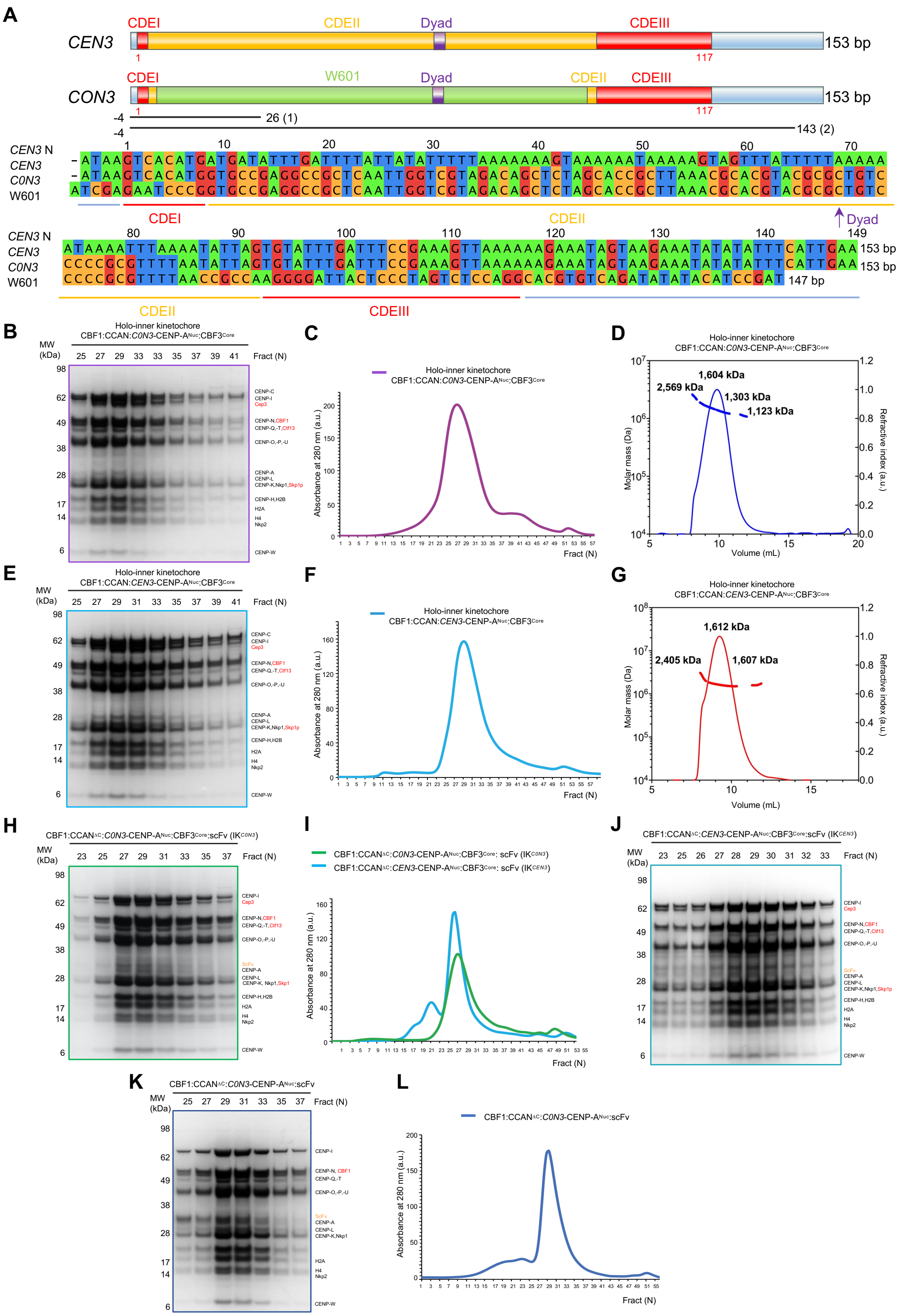

Fig. S1. Reconstituted inner kinetochore complexes. (**A**) Top: Schematic of the native *CEN3* sequence (top) and chimeric *C0N3* sequence used in this study. In the *C0N3* sequence, a large part of CDEII is replaced by the W601 sequence to increase nucleosome stability. The regions of *C0N3* that interacts with CBF1:CCAN-*C0N3* and the inner

kinetochore (IK^{C0N3}) are indicated below as black lines (1) and (2), respectively. Below: Alignment of DNA sequences used to reconstitute native 153 bp *CEN3*-CENP-A^{Nuc} (*CEN3*) and the near-native 153 bp *C0N3*-CENP-A^{Nuc} (*C0N3*) used in this study. The W601 sequence (*95*) is also shown. Alignment figure generated using Jalview (*96*). (**B**) Coomassie blue-stained SDS PAGE of the *S. cerevisiae* holo-inner kinetochore complex without stabilizing scFv and with CENP-C (CBF1:CCAN:*C0N3*-CENP-A^{Nuc}:CBF3^{Core}). (**C**) Corresponding size-exclusion chromatogram profile for sample in (B) (Agilent 1000Å column). (**D**) SEC-MALS profile of the *S. cerevisiae* holo-inner kinetochore complex. The species with mass of 1.6 MDa is consistent with a complex having a stoichiometry of 1xCBF1 dimer:2xCCAN:1x*C0N3*-CENP-A^{Nuc}:1xCBF3^{Core} (calculated molecular mass of 1.615 MDa). Dissociation of CBF3^{Core} (240 kDa) would be consistent with a proportion of molecules having a mass of 1.3 MDa. (**E**) Coomassie blue-stained SDS PAGE of the native *S. cerevisiae* holo-inner kinetochore complex without stabilizing scFv and with CENP-C (CBF1:CCAN:*CEN3*-CENP-A^{Nuc}:CBF3^{Core}). (**F**) Corresponding size-exclusion chromatogram profile for sample in (E) (Agilent 1000Å column). (**G**) SEC-MALS profile of the native (*CEN3*) *S. cerevisiae* holo-inner kinetochore complex. Calculated mass is 1.615 MDa. **(H)** Coomassie blue-stained SDS PAGE of CBF1:CCAN$^{\Delta C}$:*C0N3*-CENP-A^{Nuc}:CBF3^{Core}:scFv (inner kinetochore (IK^{C0N3})). (**I**) Comparative size-exclusion chromatogram profiles for samples in (H) and (J) (Agilent 1000Å column). (**J**) Coomassie blue-stained SDS PAGE of CBF1:CCAN$^{\Delta C}$:*CEN3*-CENP-A^{Nuc}:CBF3^{Core}:scFv (IK^{CEN3})). (**K**) Coomassie blue-stained SDS PAGE of CBF1:CCAN$^{\Delta C}$:*C0N3*-CENP-A^{Nuc}:scFv. (**L**) Corresponding size-exclusion chromatogram profile for sample in (K) (Agilent 1000Å column).

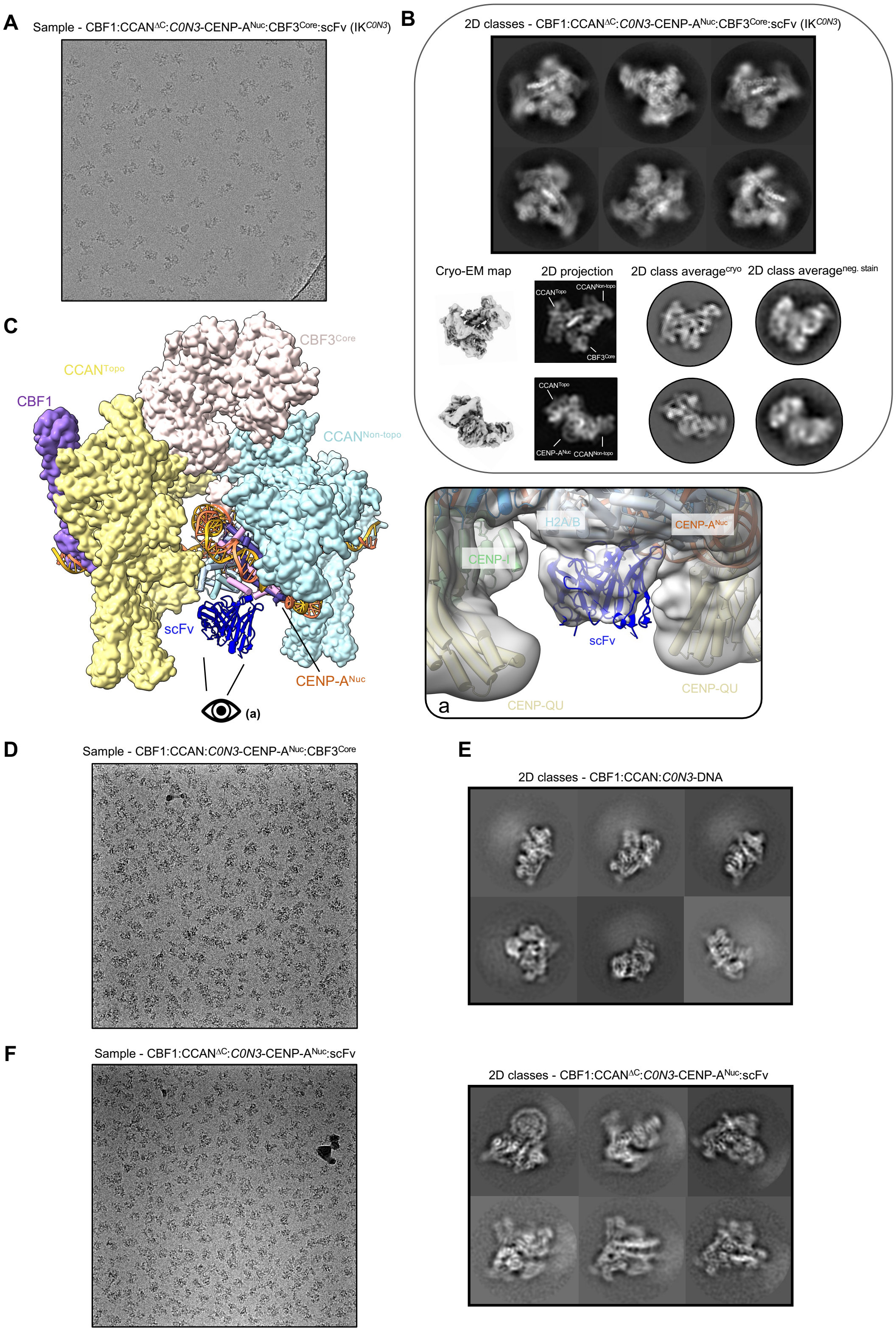

Fig. S2. Micrographs and 2D class averages of IK (sub)assembly structures determined in this study. (A) Representative micrograph of the inner kinetochore (IK^{C0N3}). **(B)** Top: 2D class averages of the inner kinetochore (IK^{C0N3}). Bottom: From left to right, oriented consensus cryo-EM maps of IK^{C0N3}, 2D projections of consensus map,

experimental cryo-EM 2D class averages matching the 2D projections, corresponding experimental negative stain 2D class averages. In the top set of views, CCAN^{Topo}, CCAN$^{Non\text{-}topo}$, CENP-A^{Nuc} and CBF3^{Core} are visible. In the bottom set of views CBF3^{Core} is obscured. (**C**) In the inner kinetochore complex (IK^{C0N3}) a single scFv is docked on a free face of CENP-A^{Nuc}, no contacts between scFv and any CCAN modules were observed in the cryo-EM density or model (inset a – right panel). (**D**) Representative micrograph of the holo-inner kinetochore complex: CBF1:CCAN:*C0N3*-CENP-A^{Nuc}:CBF3^{Core}. (**E**) 2D classes calculated for CBF1:CCAN:*C0N3*-DNA from the CBF1:CCAN:*C0N3*-CENP-A^{Nuc}:CBF3^{Core} cryo-EM data set. (**F**) Representative micrograph and 2D class averages of CBF1:CCAN$^{\Delta C}$:*C0N3*-CENP-A^{Nuc}:scFv (no CBF3^{Core}).

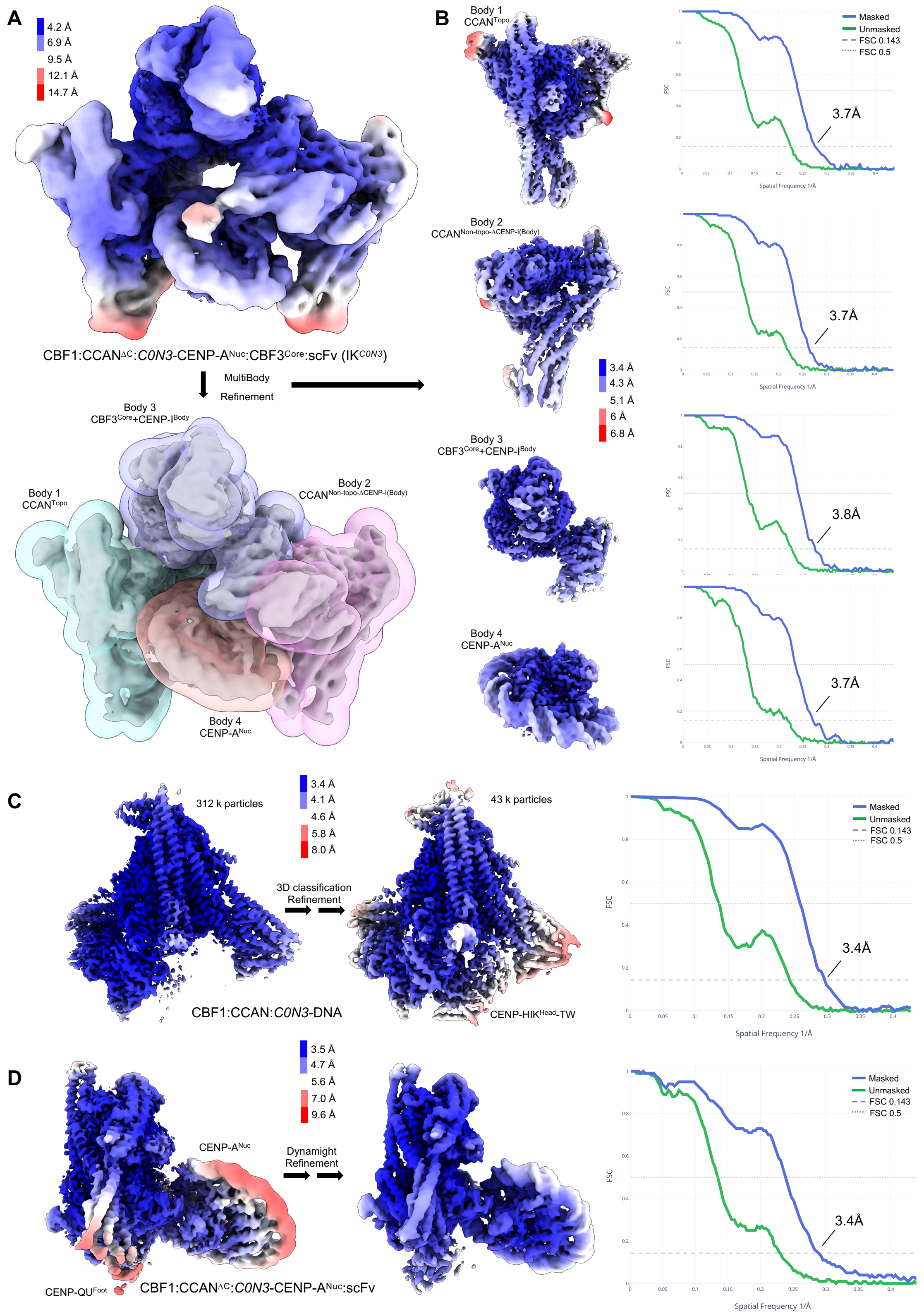

Fig. S3. Local resolution maps and FSC curves of complexes determined in this study. Local resolution estimates and FSC curves of the three reconstructions presented in this paper. (**A**) 108,672 particles contributed to a ~5 Å resolution consensus reconstruction of the inner kinetochore complex - CBF1:CCAN$^{\Delta C}$:*C0N3*-CENP-

A^{Nuc}:CBF3^{Core}:scFv (IK^{C0N3}). (**B**) Multibody refinement with four bodies (CCAN^{Topo}, CCAN$^{Non\text{-}topo\text{-}\Delta CENP\text{-}I\text{-}Body}$, CBF3^{Core}+CENP-I^{Body} and CENP-A^{Nuc}) increased the resolution to 3.7-3.8 Å for each body. (**C**) A consensus refinement of 311,873 CBF1:CCAN:*C0N3*-DNA particles resulted in a 3.4 Å resolution reconstruction with diffuse density for CENP-HIK^{Head}-TW. Extensive masked 3D classification and subsequent refinement resulted in a 3.4 Å reconstruction, from 43,467 particles where side-chains were resolved for CENP-HIK^{Head}-TW. (**D**) A consensus refinement of 100,311 CBF1:CCAN:*C0N3*-CENP-A^{Nuc}:scFv particles resulted in a 3.7 Å resolution reconstruction with well-defined density for CCAN while the CENP-A^{Nuc} and CENP-QU^{Foot} modules were resolved at significantly lower resolution. After deformation refinement with Dynamight (Materials and Methods) the local resolutions of CENP-A^{Nuc} and CENP-QU^{Foot} increased significantly, as well as the global resolution, now at 3.4 Å. At this improved resolution the model major and minor grooves of the *C0N3* DNA duplex were resolved, allowing for accurate mapping of the dyad.

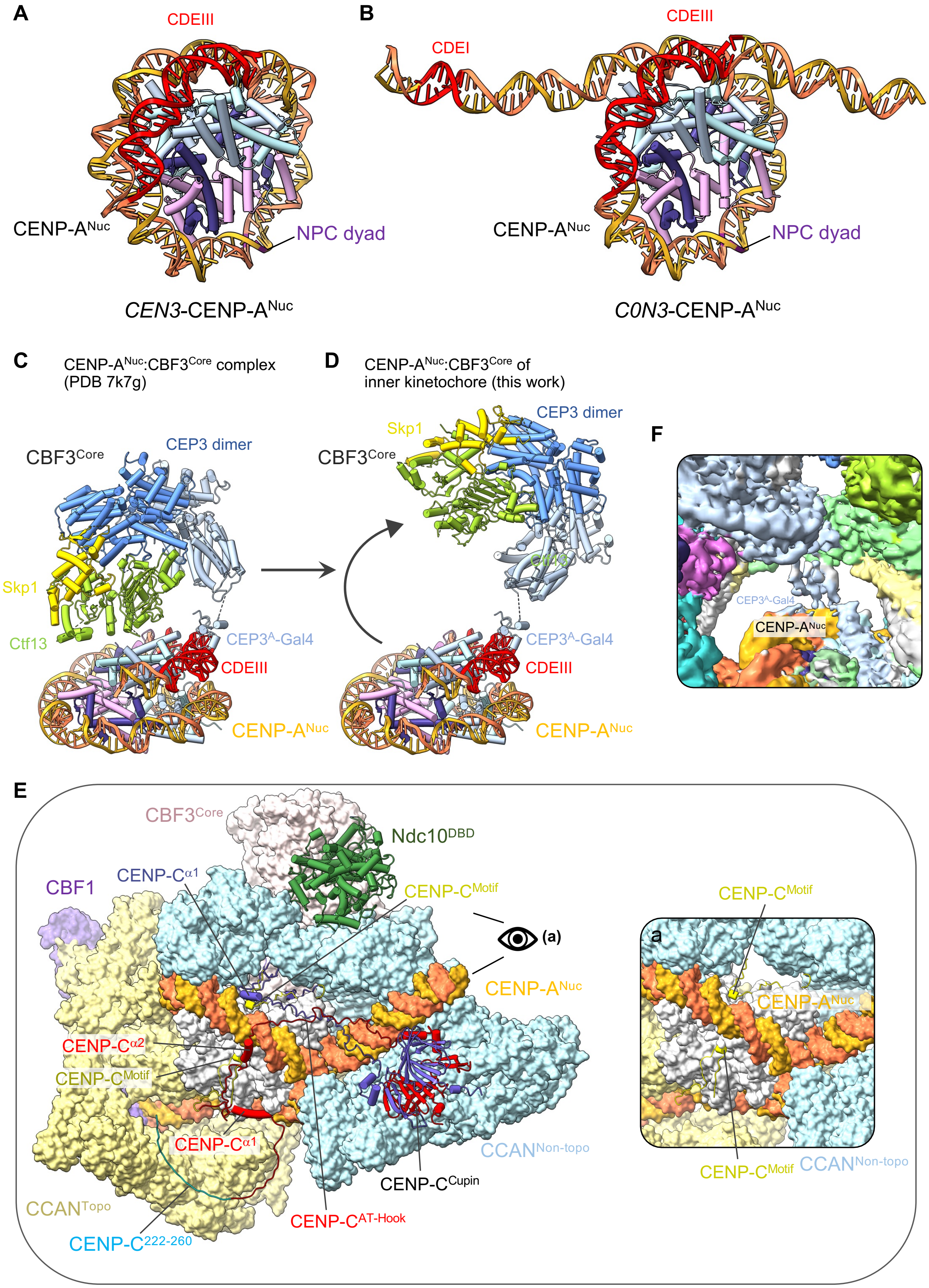

Fig. S4. Structures of CBF3^{Core} and model of inner kinetochore-CENP-A^{Nuc} complex with CENP-C and Ndc10^{DBD}. (**A**) and (**B**). Comparison of *CEN3*-CENP-A^{Nuc} (*36*) (A) with *C0N3*-CENP-A^{Nuc} as part of the inner

kinetochore from this study (B). CDEI (disordered in the *CEN3*-CENP-A^{Nuc} structure) is exposed in the inner kinetochore due to unwrapping of the 5'end. Likewise, the 3'end is slightly more unwrapped in the inner kinetochore compared to the *CEN3*-CENP-A^{Nuc} and engaged by $CCAN^{Non\text{-}topo}$. (**C**) and (**D**) Comparison of the binding mode of $CBF3^{Core}$ to CENP-A^{Nuc} in isolation (*36*) (C), and in the context of the inner kinetochore (this work) (D). $CBF3^{Core}$ rotates around the flexible hinge linking it to the Gal4 domain when in the context of the inner kinetochore. (**E**) A model showing the inner kinetochore-CENP-A^{Nuc} complex represented by a surface and the CENP-C dimer (residues 222 to C-term) and $Ndc10^{DBD}$ (DNA-binding domain of Ndc10, residues 1-540) in cartoon. $Ndc10^{DBD}$ was modeled based on the cryo-EM structure of $CBF3^{Holo}$ (*50*) (PDB 6GYP). The model of CENP-C is based on the crystal structure of *S. cerevisiae* CENP-C^{Cupin} (*54*) and the cryo-EM structure of the CENP-C motif in complex with CENP-A^{Nuc} (PDB 7ON1), combined with the AlphaFold2 prediction of *S. cerevisiae* CENP-C (*55*). Regions of CENP-C linking its MIND-binding motif with residue 260 are predicted to be disordered. Residues 1 to 221 are not shown. In the model, residues 327-340 are predicted to form a basic α-helix (CENP-$C^{\alpha 2}$) that inserts into the DNA major groove of CDEII. This α-helix is within the defined DNA-binding domain (residues 256-356), and is consistent with data that CENP-C interacts with a dyad-adjacent site within CDEII (*80*). The CENP-C motif (residues 282-305), is shown in yellow. Residues 222-240 of CENP-C are in close proximity to the CENP-$Q^{192\text{-}220}$ α-helix. These regions of CENP-C and CENP-Q were shown to interact by CLMS (*40*), and deletion of either region abolished phospho-CENP-C association with CENP-QU (*97*). The CENP-LN and CENP-HIKM-binding motifs of human CENP-C (*42, 81, 98-100*) are not conserved in *S. cerevisiae* CENP-C/Mif2p. Likewise, the CENP-C-binding sites on human CENP-LN and CENP-HIKM (*42, 81, 100*) are not present in *S. cerevisiae* CCAN. The Ndc10 component of CBF3 only weakly associates with $CBF3^{Core}$ (*36*), and its inclusion in our inner kinetochore reconstitution resulted in heterogeneous complexes. However, docking Ndc10 onto the inner kinetochore, guided by the $CBF3^{Holo}$ structure (*50*), indicated a position that generates interfacial contacts with both CENP-I and CENP-L of $CCAN^{Non\text{-}topo}$. Inset a: close up of the CENP-C motifs bound to the nucleosome faces in the context of the inner kinetochore. The top CENP-C motif is not sterically hindered by CENP-HIK^{Head}-TW of $CCAN^{Non\text{-}topo}$, the bottom CENP-C motif is displaced by the scFv antibody in our reconstituted inner kinetochore. (**F**) Close-up of the $CEP3^{A}$-Gal4 density in the cryo-EM map at lower threshold.

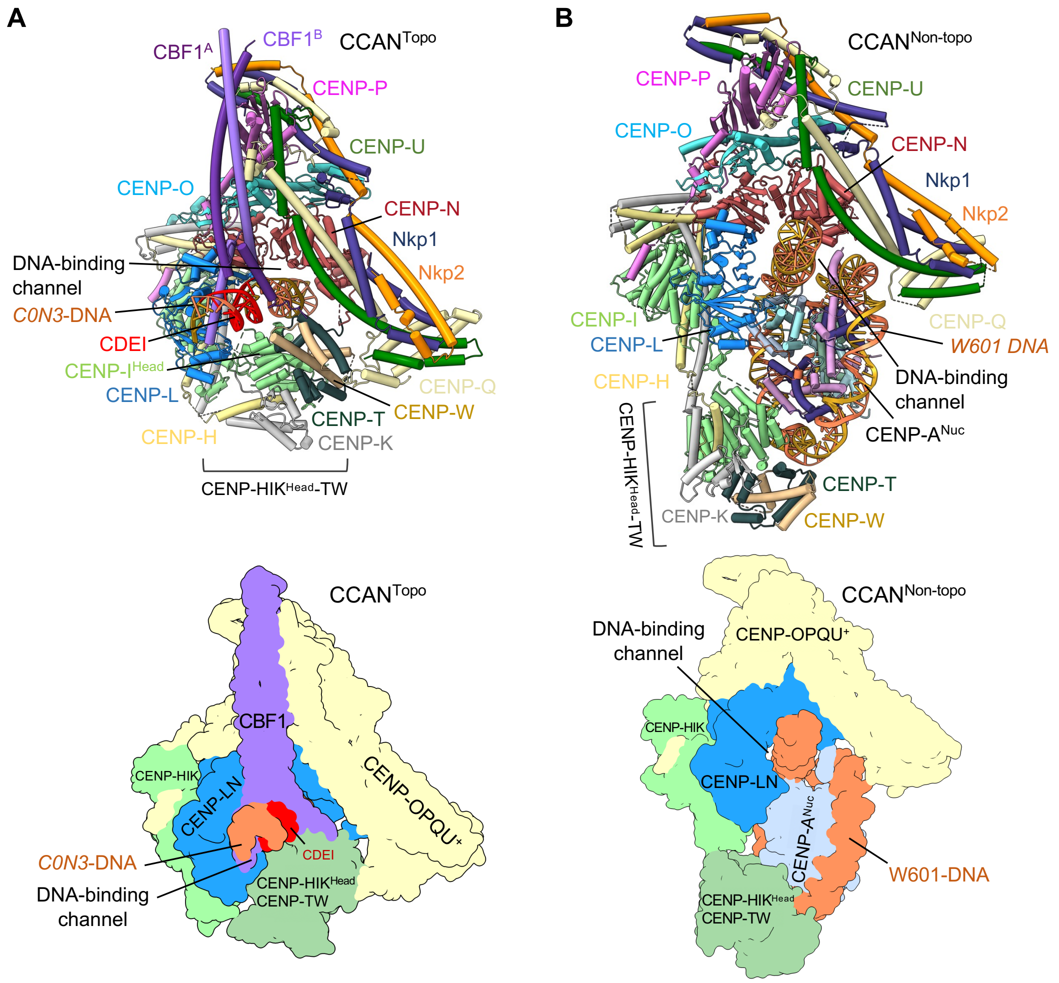

Fig. S5. Overall architecture of CBF1:CCAN^{Topo} and CCAN$^{Non\text{-}topo}$. (A) Ribbons representation of the CBF1:CCAN:DNA complex with components annotated and a schematic below. The CENP-HIK^{Head}-TW module topologically entraps the *CEN3* DNA within the CENP-LN DNA-binding channel. The CENP-HIK^{Head}-TW module adopts an 'upwards' conformation, contacting the DNA duplex. (**B**) Structure of CCAN:*W601*-CENP-A^{Nuc} complex with CENP-A^{Nuc} reconstituted with W601 DNA, in the absence of CBF1. Schematic below. From (*34*). CENP-HIK^{Head}-TW module adopts a 'downwards' conformation, interacting with the CENP-A^{Nuc} DNA gyre.

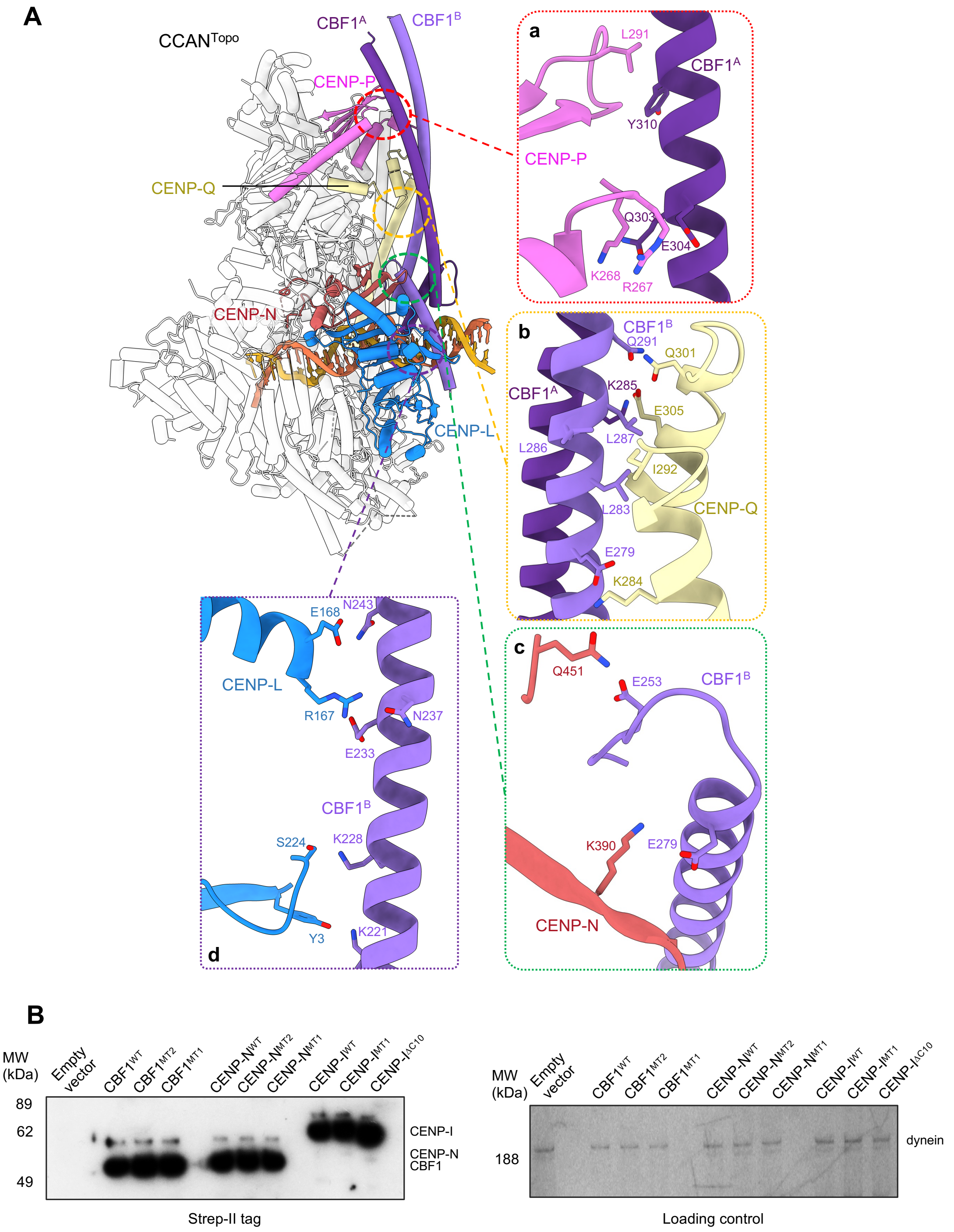

Fig. S6. (A) CBF1 interacts with the back-face of CCAN^Topo^. Details of how CBF1 interacts with CENP-P (inset a), CENP-Q (inset b), CENP-N (inset c) and CENP-L (inset d). (**B**) Western blot showing that wild type and mutant CBF1, CENP-N and CENP-I are expressed at equivalent levels in the BJ2168*^CEN3^* strain (left) and loading control (right; Coomassie-blue-stained gel shows dynein).

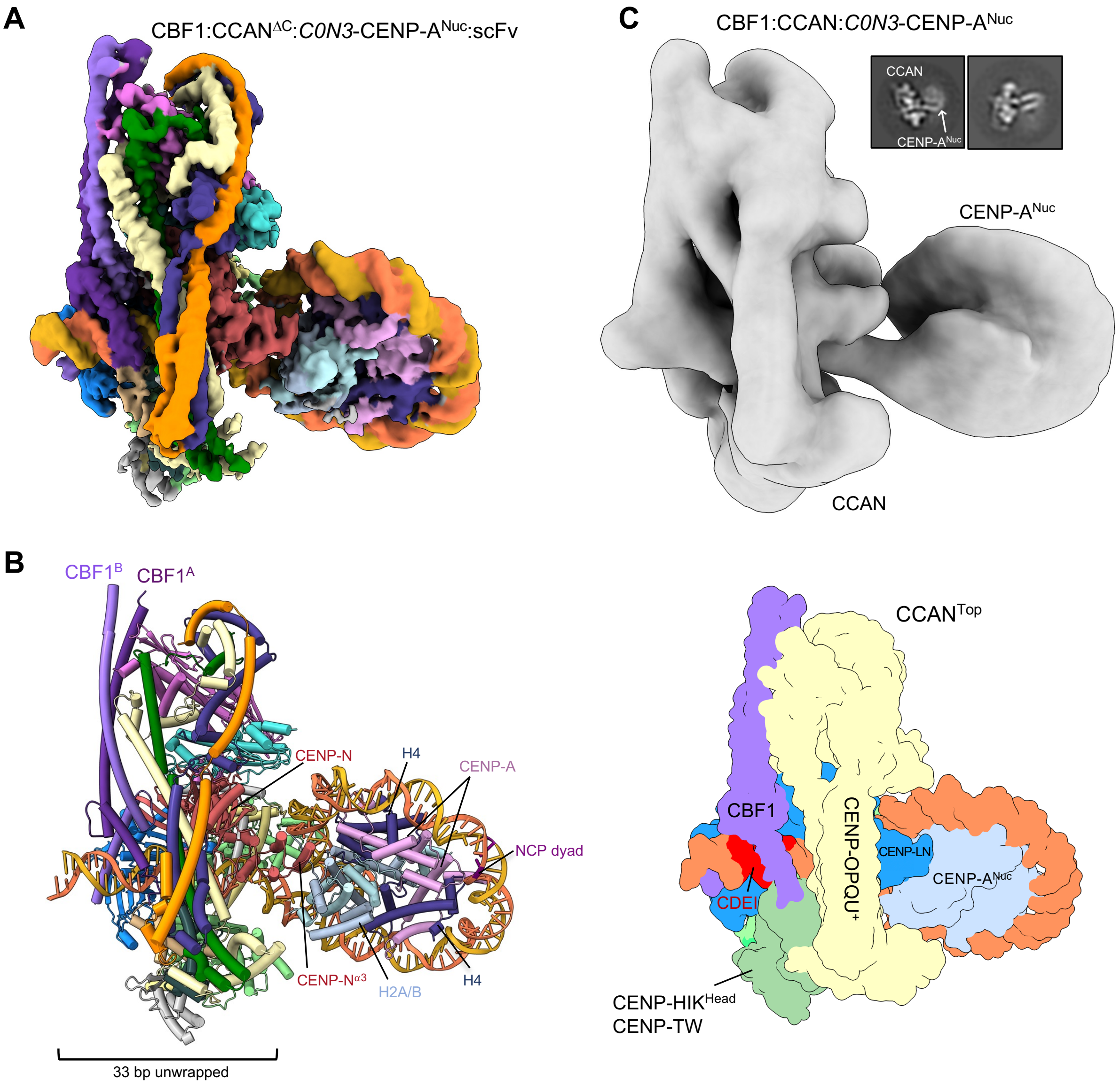

Fig. S7. Structure of the CBF1:CCAN:CENP-A^{Nuc} complex (**A**) Cryo-EM map of CBF1:*C0N3*-CCAN$^{\Delta C}$:CENP-A^{Nuc}:scFv, i.e., stabilized in the presence of scFv without CBF3^{Core}, at 3.4 Å resolution, showing how CBF1:CCAN^{Topo} topologically entraps the unwrapped 5'-end of *C0N3*-CENP-A^{Nuc}. (**B**) Ribbons representation of the CBF1:CCAN:CENP-A^{Nuc} complex. Schematic on the right. (**C**) Cryo-EM map of CBF1:CCAN:*C0N3*-CENP-A^{Nuc} (from the CBF1:CCAN:*C0N3*-CENP-A^{Nuc}:CBF3^{Core} cryo-EM data set - with CENP-C and no scFv) at 12 Å resolution. 11,435 particles were used for this reconstruction (0.75% of initial particles extracted). 2D classes show different projections of the monomeric CBF1:CCAN:CENP-A^{Nuc} complex: 'face on' and 'side' views of the CENP-A^{Nuc} density.

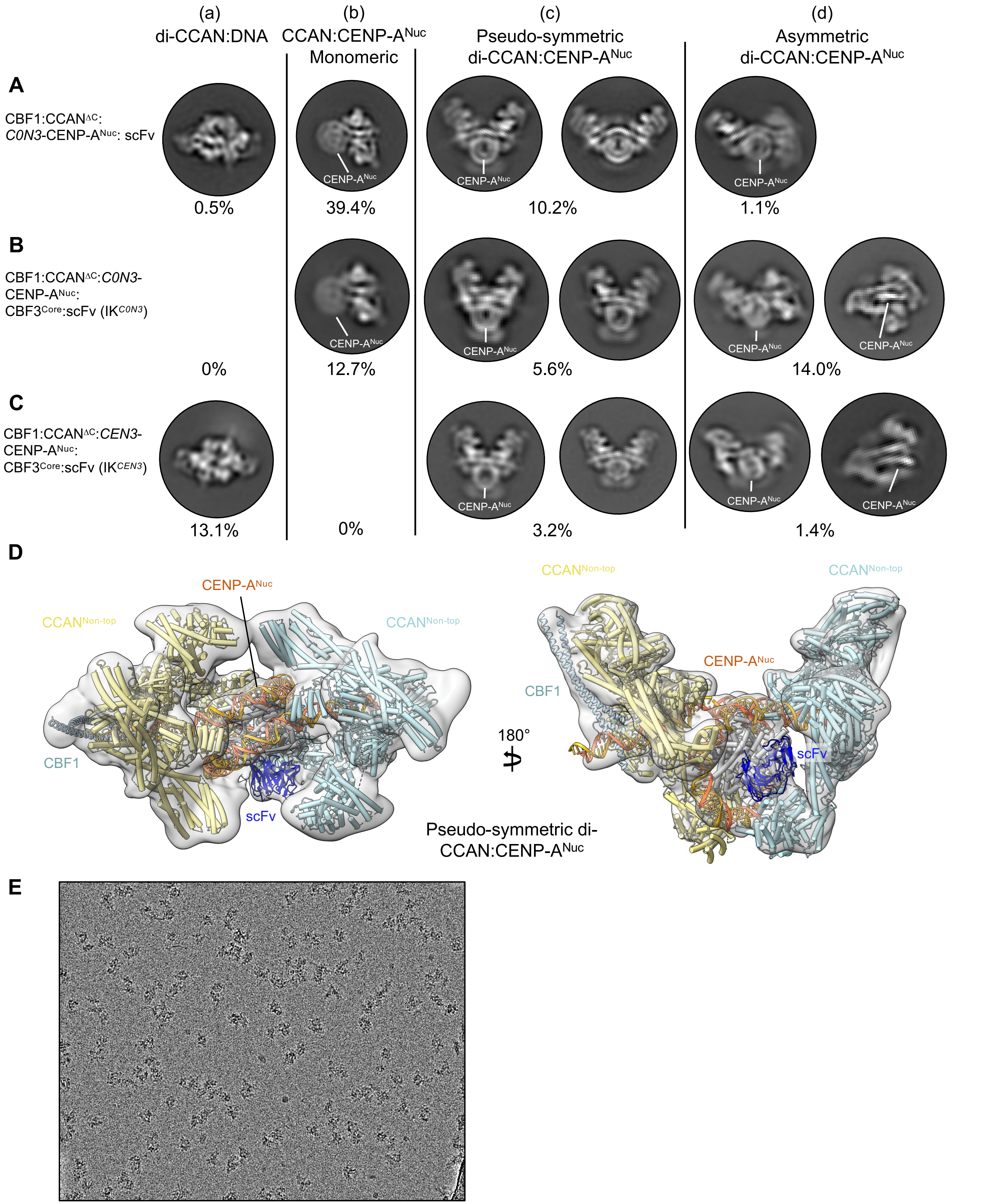

Fig. S8. Gallery of 2D classes representing the structural states of CCAN species. (**A-C**) 2D class averages from cryo-EM data sets for (**A**) CBF1:CCAN$^{\Delta C}$:*C0N3*-CENP-A^{Nuc}:scFv, (**B**) CBF1:CCAN$^{\Delta C}$:*C0N3*-CENP-A^{Nuc}:CBF3^{Core}:scFv (IK^{C0N3}) and (**C**) CBF1:CCAN$^{\Delta C}$:*CEN3*-CENP-A^{Nuc}:CBF3^{Core}:scFv (IK^{CEN3}). Shown are four sets of 2D class averages represented in the three data sets: (a) Dimeric CCAN (DNA, no CENP-A^{Nuc}), similar to the apo-dimeric CCAN reported by (*101*), (b) monomeric CCAN:CENP-A^{Nuc}, (c) pseudo-symmetric and (d) asymmetric di-CCAN configurations assembled on CENP-A^{Nuc}. (**A**) In the CBF1:CCAN$^{\Delta C}$:*C0N3*-CENP-A^{Nuc}:scFv dataset, only ~0.5% of the particles correspond to a dimeric CCAN:DNA species,

devoid of CENP-A^{Nuc} (a), while ~40% of the particles correspond to monomeric CBF1:CCAN:*C0N3*-CENP-A^{Nuc} with a CCAN^{Topo} configuration (b). ~10% of particles correspond to pseudo-symmetric di-CCAN$^{\text{Non-topo}}$ assembled on CENP-A^{Nuc} (c). Only ~1% of particles correspond to an asymmetric di-CCAN species assembled on CENP-A^{Nuc}, with one CCAN^{Topo} and one CCAN$^{\text{Non-topo}}$. (**B**) In the CBF1:CCAN$^{\Delta\text{C}}$*:C0N3*-CENP-A^{Nuc}:CBF3^{Core}:scFv (IK^{C0N3}) sample, no dimeric CCAN:DNA species were observed (a), and significantly less monomeric CBF1:CCAN$^{\Delta\text{C}}$:*C0N3*-CENP-A^{Nuc} (b) and pseudo-symmetric di-CCAN$^{\text{Non-topo}}$ assembled on CENP-A^{Nuc} (c). Instead, the equilibrium is shifted towards the asymmetric di-CCAN species assembled on CENP-A^{Nuc} of the inner kinetochore (~14% of particles) (d). (**C**) In the CBF1:CCAN$^{\Delta\text{C}}$:*CEN3*-CENP-A^{Nuc}:CBF3^{Core}:scFv (IK^{CEN3}) sample, ~13% of particles correspond to a dimeric CCAN:DNA species, devoid of CENP-A^{Nuc} (a), while only 3.2% of particles correspond to the pseudo-symmetric di-CCAN$^{\text{Non-topo}}$ species assembled on CENP-A^{Nuc} (c), and 1.4% correspond to asymmetric di-CCAN species as observed in the inner kinetochore (d). No monomeric CCAN:CENP-A^{Nuc} species were observed (b). These numbers are indicative of poor *CEN3*-CENP-A^{Nuc} stability in solution and/or on cryo-EM grids. The CCAN modules in the pseudo-symmetric di-CCAN species adopt a range of conformational states with respect to CENP-A^{Nuc} from more closed to more open, as is apparent from the 2D class averages in column (c). Particle fractions are calculated as a percentage of total extracted particles per dataset. The remaining extracted particles were denatured and/or false positives. (**D**) A 10 Å-resolution 3D reconstruction of a pseudo-symmetric di-CCAN:*CEN3*-CENP-A^{Nuc}:CBF1 assembly (IK^{CEN3}), where both CCAN protomers bind the *CEN3* DNA non-topologically in the absence of CBF3^{Core}. scFv binds to one face of CENP-A^{Nuc}. An identical reconstruction was obtained from the CBF1:CCAN$^{\Delta\text{C}}$*:C0N3*-CENP-A^{Nuc}:CBF3^{Core}:scFv (IK^{C0N3}) cryo-EM data set. (**E**) Representative micrograph of the inner kinetochore (IK^{CEN3}) CBF1:CCAN$^{\Delta\text{C}}$:*CEN3*-CENP-A^{Nuc}:CBF3^{Core}:scFv complex.

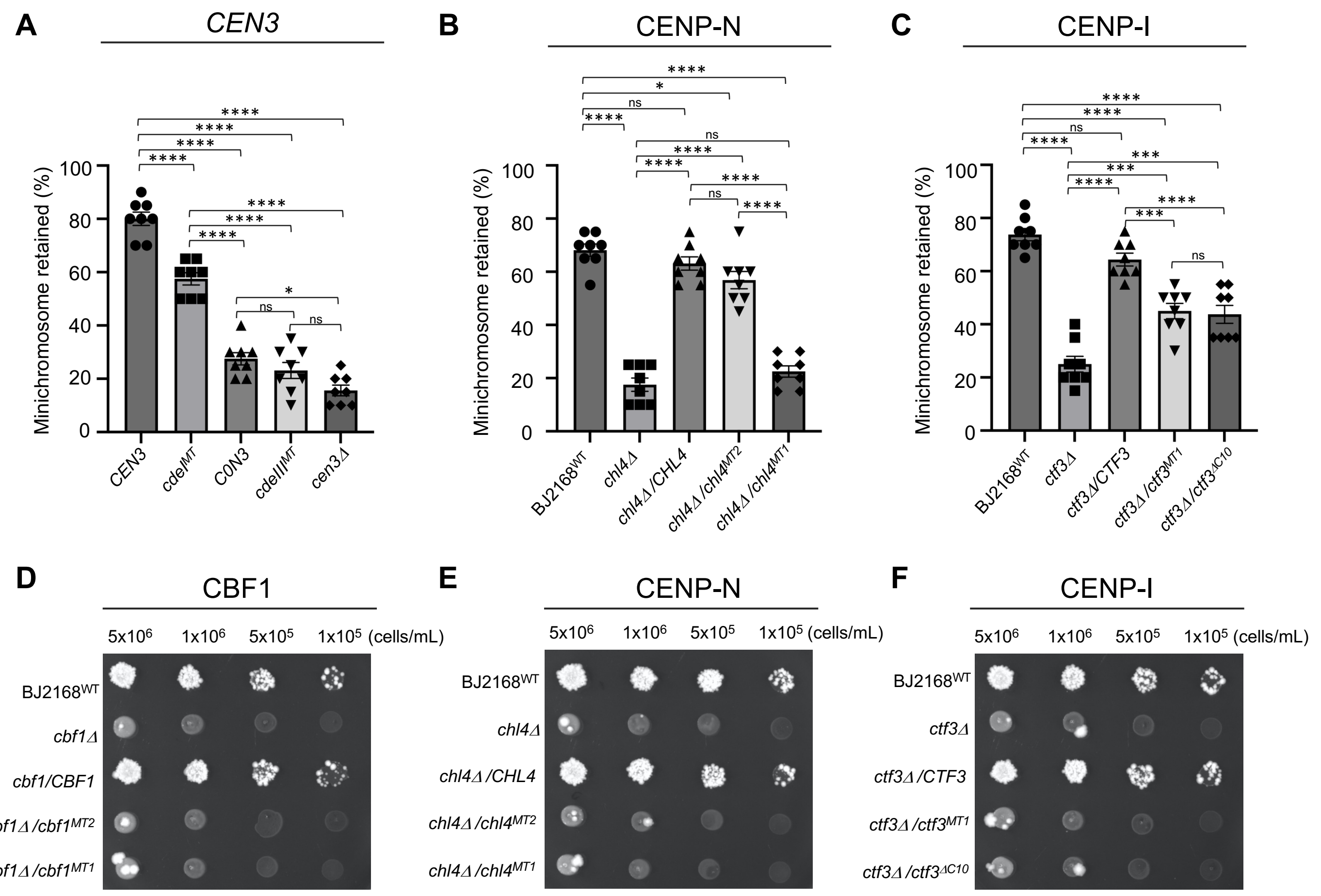

Fig. S9. *In vivo* testing of the inner kinetochore structure. (**A-C**) Quantification of chromosome segregation loss for (**A**) *CEN3* mutants. *CEN3*: minichromosome with wild type *CEN3*; *cdeI*^{MT}: CDEI mutant - GTCACATG to AATTGGCT; *C0N3*: minichromosome with *CEN3* replaced with *C0N3*; *cdeIII*^{MT}: CDEIII mutant - CCG to AGC (Gal4-DNA-binding motif of CBF3 (*22, 23, 50*)). *cen3Δ*: minichromosome with *CEN3* deleted; *: p=0.0125, ****: p<0.0001, ns: (not significant) p>0.2. (**B**) *CHL4* (CENP-N) mutants: *chl4*^{MT1} (DNA-binding groove: *chl4*^{K22S,K26S,R67S,K100S,K103S,K105S,R198S,K217S,K245S,K249S,K384S,K401S,K403S}), *chl4*^{MT2} (histone H2A-H2B-binding: *chl4*^{D48R,D50R,E56R,E63R}); *: p=0.029, ****: p<0.0001, ns: p>0.4. (**C**) *CTF3* (CENP-I) mutants: *ctf3*^{MT1} (DNA binding: *ctf3*^{R215S,K216S,K219S,R222S,K225S}), *ctf3*$^{\Delta C10}$ (CBF3 binding: *ctf3*$^{F719S,\Delta 724\text{-}733}$); ***: 0.0001<p>0.0003, ****: p<0.0001, ns: p>0.15. All experiments were performed independently eight times (i.e. *N*=8 biological replicates), N=20 (sample size) per experiment. Data were analyzed using Prism 9 (ver 9.5.1, GraphPad). Data in all groups (wild type and associated mutants) in each of the three data sets were included in a family-wise comparison analysis using the ordinary one-way ANOVA Tukey's multiple comparisons test (10 comparisons/family). The corresponding adjusted p values are indicated. The mean is indicated for each group. Error bars denote SEM. Data are presented as a scatter dot plot. (**D-F**) Benomyl sensitivity spot assays for yeast strains harboring mutations of, (**D**) *CBF1*: *cbf1*^{MT1} (CENP-QU-binding mutant: *cbf1*^{L283E,L287W}), *cbf1*^{MT2} (DNA-binding: *cbf1*$^{K224S/K228S/R234S/R235S/K256S}$), (**E**) CHL4 (CENP-N): *chl4*^{MT1} and *chl4*^{MT2}, and (**F**) CTF3 (CENP-I): *ctf3*^{MT1} and *ctf3*$^{\Delta C10}$, show sensitivity to benomyl.

A

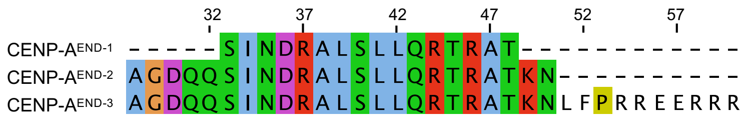

B

Peptide	N	Kd (μM)	ΔH (kcal.mol^{-1})	-TΔS (kcal.mol^{-1})	ΔG (kcal.mol^{-1})	[protein] (mM)	[peptide] (mM)
CENP-A^{END-1}	0.99 ± 0.01	11.63 ± 0.19	-11.47 ± 0.05	4.84 ± 0.03	-6.62 ± 0.01	0.180	2.18
CENP-A^{END-2}	1.08 ± 0.0	0.66 ± 0.008	-12.4 ± 0.0	4.26 ± 0.14	-8.3 ± 0.01	0.066	0.87
CENP-A^{END-3}	1.09 ± 0.01	0.72 ± 0.03	-13.10 ± 0.10	4.87 ± 0.12	-8.25 ± 0.03	0.142	1.18
CENP-A$^{END-2-R37A}$	1.13 ± 0.0	9.67 ± 0.01	-10.15 ± 0.05	3.40 ± 0.05	-6.73± 0.005	0.066	1.00

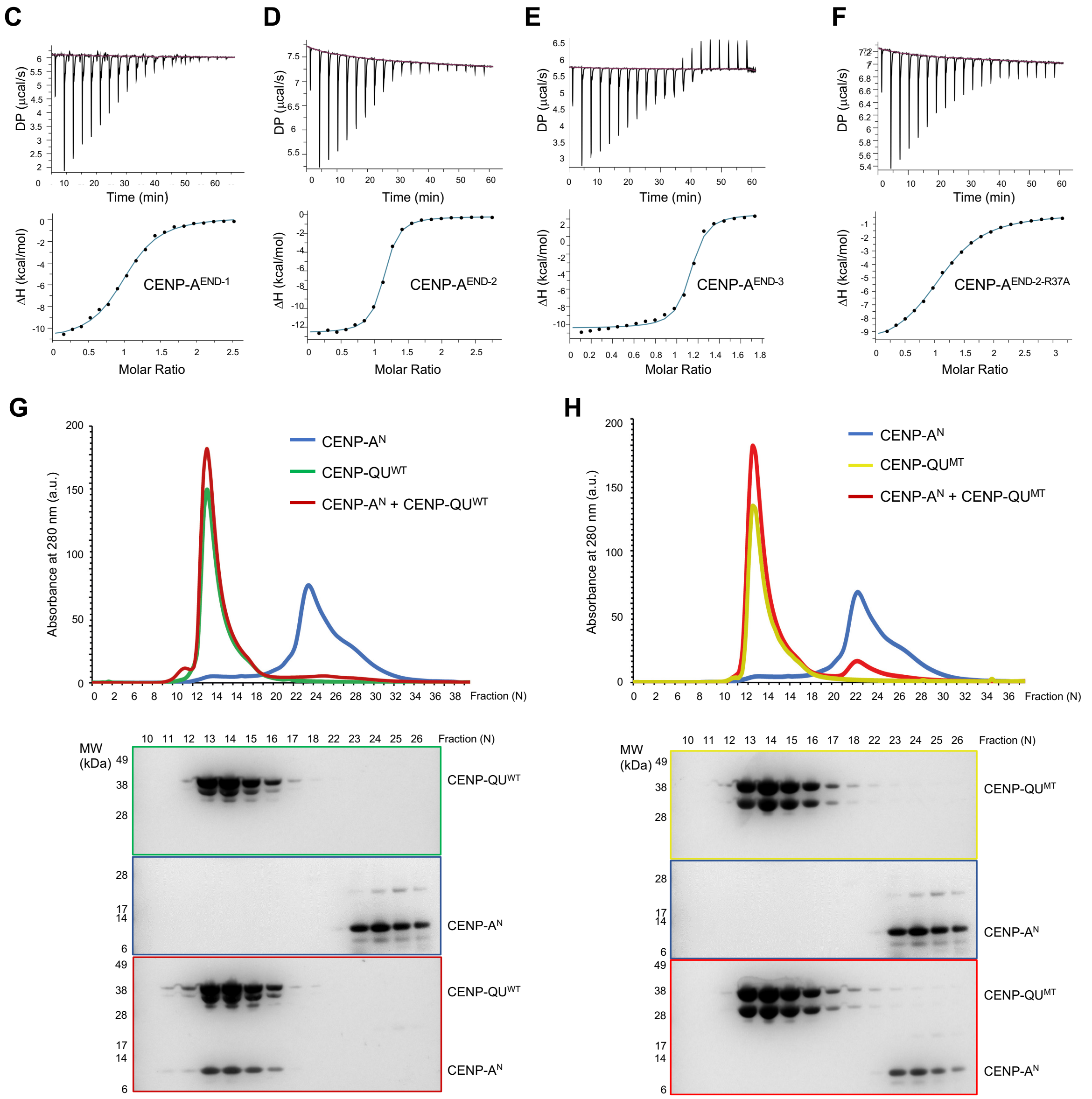

Fig. S10. The N-terminus of CENP-A (CENP-A^{N}) interacts with CENP-QU. (**A**) Sequences of CENP-A^{END} peptides modeled on the N-terminus of CENP-A used in this study. Alignment figure generated using Jalview (*96*). (**B**) Quantification of isothermal titration calorimetry data shown in panels C-E. (**C-F**) Isothermal titration calorimetry measurements of the binding of CENP-A^{END}

peptides (CENP-A^{END-1}, CENP-A^{END-2}, CENP-A^{END-3}, CENP-A$^{END-2-R37A}$, respectively) to CENP-QU. Upper panel, raw data of the titration of CENP-A^{END} into CENP-QU. Lower panel, integrated heats of injections, corrected for the heat of dilution, with the solid line corresponding to the best fit of the data using the MicroCal software. (**G**) CENP-QU and CENP-A^{N} form a stable complex, as judged by SEC. Upper panel: SEC chromatogram (Superdex S75 column), lower panel: corresponding Coomassie-blue stained PAGE. (**H**) Mutating residues in CENP-QU (D191K^{CENP-U}, D194K^{CENP-U} and E235K^{CENP-Q}) predicted to interact with R37, R46 and K49 of CENP-A^{N} disrupts the CENP-QU:CENP-A^{N} complex. Upper panel: SEC chromatogram (Superdex S75 column), lower panel: corresponding Coomassie-blue stained PAGE.

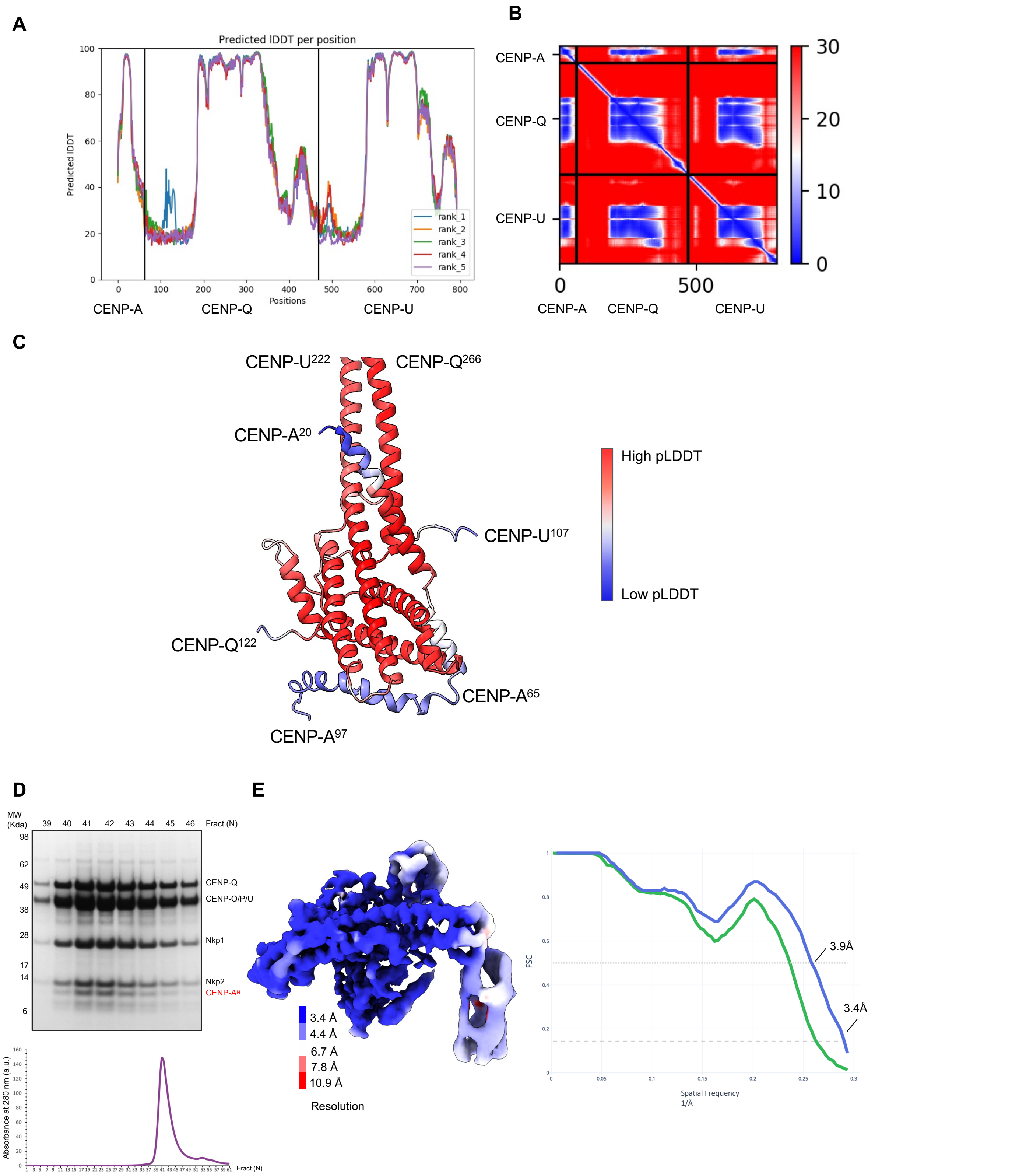

Fig. S11. Confidence metrics for the CENP-A^{N}:CENP-QU AlphaFold2 prediction. (**A**) Predicted local distance difference test (lDDT) values (*102*) for the AlphaFold2 model of CENP-A^{N}:CENP-QU. A higher score indicates higher confidence in the prediction. **(B)** The predicted alignment error (PAE) heat map for the CENP-A^{N}:CENP-QU

AlphaFold2 prediction. The PAE heat map shows the predicted error (in angstroms) between all pairs of residues, with blue indicating lower error and red indicating higher error. The PAE plot suggests high confidence for the predicted interactions between CENP-QU and CENP-A^{END}. (**C**) The pLDDT values mapped onto the predicted model of CENP-A^{N}:CENP-QU. Residues are colored in a scale of high (red) to low (blue) pLDDT values. The model of CENP-QU bound to CENP-A^{END} was predicted with high confidence (pLDDT > 90), whereas the residues 65-97 of CENP-A were predicted with relatively low confidence (pLDDT < 60). (**D**) Top panel: Coomassie blue-stained PAGE of the CENP-OPQU+:CENP-A^{N} complex, below: corresponding SEC chromatogram (Agilent 1000Å column). (**E**) Left: cryo-EM map of the CENP-OPQU+:CENP-A^{N} complex color-coded according to local resolution. Right: FSC curves of the CENP-OPQU+:CENP-A^{N} complex (green (unmasked), blue (masked)).

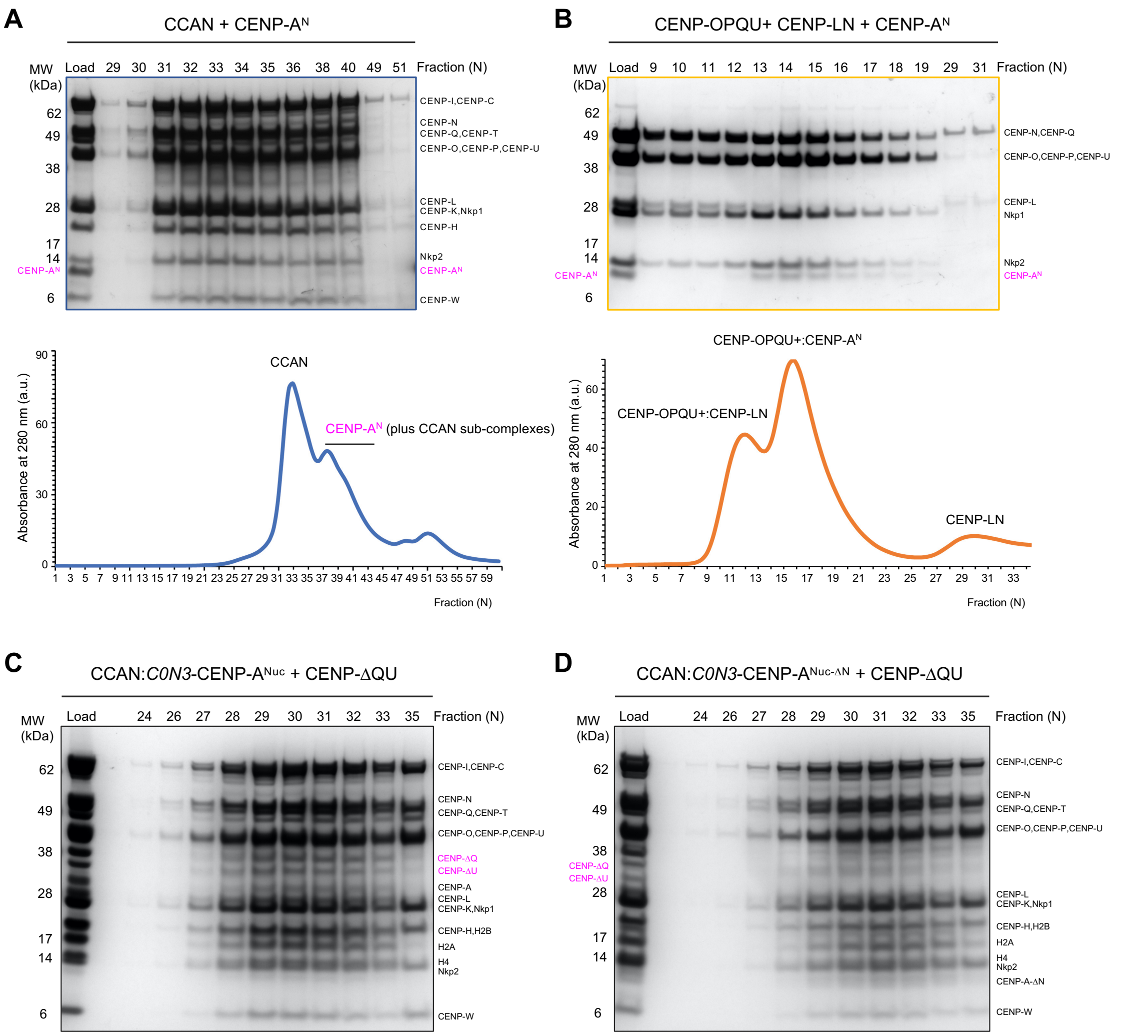

Fig. S12. CENP-A^{N}-binding to CCAN is auto-inhibited and supernumerary CENP-QU interacts with the inner kinetochore:CENP-A^{Nuc} complex through CENP-A^{N}. (**A**) CENP-A^{N} (residues 1-82) does not bind to CCAN. Upper panel: Coomassie-blue stained PAGE, lower panel: corresponding SEC chromatogram (Agilent 1000Å column). (**B**) The binding of CENP-A^{N} to CENP-OPQU+ is displaced by CENP-LN. In the presence of CENP-A^{N} and CENP-LN, CENP-OPQU+ forms either CENP-OPQU+:CENP-A^{N} or CENP-OPQU+:CENP-LN complexes, showing that CENP-A^{N} and CENP-LN binding to CENP-OPQU+ is mutually exclusive. Upper panel: Coomassie-blue stained PAGE, lower panel: corresponding SEC chromatogram (Superose 6 column). (**C**) Coomassie-blue stained PAGE of SEC fractions (Agilent 1000Å column) showing binding of supernumerary CENP-QU to the inner kinetochore:CENP-A^{Nuc} complex. The addition CENP-ΔQU complex (CENP-ΔQ, CENP-ΔU) consisted of residues 30-255 (CENP-ΔQ) and 1-294 (CENP-ΔU) to distinguish from CENP-QU assembled into CCAN. (**D**) Binding of supernumerary CENP-QU is abolished when CENP-A$^{\Delta N}$ (deletion of CENP-A residues 1-129) was used to reconstitute CENP-A^{Nuc} (CENP-A$^{Nuc-\Delta N}$). Coomassie-blue stained PAGE of SEC fractions (Agilent 1000Å column).

Table S1 Nomenclature and organization of kinetochore subunits and sub-complexes

Holo-inner kinetochore: CBF1:CCAN:*CEN3*-CENP-A^{Nuc}:CBF3^{Core} and CBF1:CCAN:*C0N3*-CENP-A^{Nuc}:CBF3^{Core} (1.615 mDa)
Inner kinetochore (IK^{CEN3}): CBF1:CCAN$^{\Delta C}$:*CEN3*-CENP-A^{Nuc}:CBF3^{Core}:scFv
Inner kinetochore (IK^{C0N3}): CBF1:CCAN$^{\Delta C}$:*C0N3*-CENP-A^{Nuc}:CBF3^{Core}:scFv

Subunit	*S.c.* name	Length	Mol mass kDa	[a]Stoichio-metry	Sub-complex	Level2	Level3
***C0N3*- and *CEN3*-CENP-A^{Nuc}**					**CENP-A^{Nuc}**	**Inner kinetochore**	**Holo-kinetochore**
**CENP-A**	Cse4	229	26.8	2			
**H2A**	HTA2	132	14.0	2			
**H2B**	HTB1	131	14.2	2			
**H4**	HHF1	103	11.4	2			
***CEN3* DNA**		153 bp	94.9	[b]1			
***C0N3* DNA**		153 bp	94.9	[b]1			
**CENP-C**					**CENP-C**		
**CENP-C**	Mif2	549	62.5	2			
**CENP-HIK-TW complex**					**CCAN**		
**CENP-H**	Mcm16	181	21.1	2			
**CENP-I**	Ctf3	733	84.3	2			
**CENP-K**	Mcm22	239	27.6	2			
**CENP-T**	Cnn1	361	41.3	2			
**CENP-W**	Wip1	89	10.2	2			
**CENP-LN complex**							
**CENP-L**	Iml3	245	28.1	2			
**CENP-N**	Chl4	458	52.7	2			
**CENP-OPQU+/COMA+ complex**							
**CENP-O**	Mcm21	368	43.0	2			
**CENP-P**	Ctf19	369	42.8	2			
**CENP-Q**	Okp1	406	47.3	2			
**CENP-U**	Ame1	324	37.5	2			
**Nkp1**	Nkp1	238	27.0	2			
**Nkp2**	Nkp2	153	17.9				
				2			
**CBF3^{Core} complex**					**CBF3**		
**Cep3**	Cep3	608	71.4	2			
**Ctf13**	Ctf13	478	56.3	1			
**Skp1**	Skp1	194	22.3	1			
**Ndc10**	Ndc10	956	111.9				
**CBF1 complex**					**CBF1**		
**Cbf1**	Cbf1	351	39.4	2			
**Ndc80 complex**					**NDC80c**	**KMN network of outer kinetochore**	
**Ndc80**	Ndc80	691	80.5				
**Nuf2**	Nuf2	451	53.0				
**Spc24**	Spc24	213	24.6				
**Spc25**	Spc25	221	25.2				
**MIND/MIS12 complex**					**MIND**		
**Dsn1**	Dsn1	576	65.7				
**Nnf1**	Nnf1	201	23.6				
**Mis12**	Mtw1	289	33.2				
**Nsl1**	Nsl1	216	25.4				
**KNL1 complex**					**KNL1c**		
**Knl1**	Spc105	917	104.8				
**Zwint1**	Kre28	385	44.7				

[a]Subunit stoichiometry for individual holo-inner kinetochore complexes defined in this study.
[b]For IK^{CEN3} and IK^{C0N3}, respectively.

Table S2 Cryo-EM data collection, refinement and validation statistics

**Complex**	**CBF1:CCAN$^{\Delta C}$: *C0N3*-CENP-A^{Nuc}: CBF3^{Core}:ScFv (IK^{C0N3})**	**CBF1:CCAN: *C0N3***	**CBF1:CCAN$^{\Delta C}$: *C0N3*-CENP-A^{Nuc}: ScFv**	**CENP-OPQU+: CENP-A^{N}**
**Accession numbers**	**(EMD-17227) (PDB 8OW1)**	**(EMD-17224) (PDB 8OVW)**	**(EMD-17226) (PDB 8OW0)**	**(EMD-17225) (PDB 8OVX)**
**Data collection and processing**				
Magnification	105,000	105,000	105,000	105,000
Voltage (kV)	300	300	300	300
Electron exposure (e–/Å^2)	40	40	40	40
Detector	Falcon4	Falcon4	K3	K3
Defocus range (μm)	1.2-2.6	1.2-2.6	1.2-2.6	0.5-2.0
Pixel size (Å)	1.08	1.17	0.853	0.826
Symmetry imposed	C1	C1	C1	C1
Number of movies	10,196	8804	10,385	12,414
Initial particle images (no.)	1,217,837	1,516,955	1,142,899	3,673,108
Final particle images (no.)	108,672	43,467	100,311	595,147
Map resolution (Å)	3.7-3.8	3.4	3.4	3.4
FSC threshold	0.143	0.143	0.143	0.143
Map resolution range (Å)	3.4-6.8	3.4-8	3.4-9.6	3.4-12
Map sharpening *B* factor (Å^2)	-110	-85	-110	-70
**Refinement**				
Initial model used (PDB code)	*6QLD,6GYP*	*6QLE, ab initio*	*6QLD*	*6QLF*
Model resolution (Å)	3.7	3.4	3.5	7.0
FSC threshold	0.5	0.5	0.5	0.5
Model to map CC	0.71	0.84	0.76	0.6
Model composition				
Non-hydrogen atoms	78,805	28,484	37,995	67,63
Protein residues	8,937	3,384	4,108	885
Nucleotide	306	54	241	0
**Validation**				
B factors (Å^2)				
Protein	109.89	96.15	138.11	83.44
Nucleotide	152.19	132.94	175.66	N/A
R.m.s. deviations				
Bond lengths (Å)	0.004	0.004	0.004	0.003
Bond angles (°)	0.727	0.665	0.732	0.559
Validation				
MolProbity score	1.75	1.89	1.92	1.98
Clash score	10.49	5.45	6.04	6.73O
Poor rotamers (%)	1.00	0.10	0.41	0.28
Ramachandran plot				
Favored (%)	95.87	96.00	95.88	95.43
Allowed (%)	4.09	3.97	4.12	4.57
Disallowed (%)	0.03	0.03	0.00	0.00

Movie S1. Structure of the *S. cerevisiae* inner kinetochore on a CENP-A nucleosome. The video shows how the component sub-complexes of the inner kinetochore are arranged on a central CENP-A nucleosome to build the entire inner kinetochore-CENP-A^{Nuc} complex. The extensive unwrapping of CENP-A^{Nuc} DNA ends creates the binding sites for two CCAN protomers. The organization of $CCAN^{Topo}$ and $CCAN^{Non\text{-}topo}$ is assisted by two DNA-specific binding complexes, CBF1 and CBF3, that engage, respectively, the conserved CDEI and CDEIII sequence elements conserved in point centromeres.

Movie S2. Model for CENP-A essential N-terminal domain (CENP-A^{END}) interaction with CENP-QU. The interaction of CENP-A^{END} with CENP-QU requires that Nkp1-Nkp2 undergo a conformational change to expose the CENP-A^{END}-binding site on CENP-QU.

REFERENCES AND NOTES

1. A. Musacchio, A. Desai, A molecular view of kinetochore assembly and function. *Biology* **6**, (2017).

2. A. P. Navarro, I. M. Cheeseman, Kinetochore assembly throughout the cell cycle. *Semin. Cell Dev. Biol.*, **117**, 62–74 (2021).

3. S. Sridhar, T. Fukagawa, Kinetochore architecture employs diverse linker strategies across evolution. *Front. Cell Dev. Biol.* **10**, 862637 (2022).

4. W. C. Earnshaw, N. Rothfield, Identification of a family of human centromere proteins using autoimmune sera from patients with scleroderma. *Chromosoma* **91**, 313–321 (1985).

5. P. B. Meluh, P. Yang, L. Glowczewski, D. Koshland, M. M. Smith, Cse4p is a component of the core centromere of Saccharomyces cerevisiae. *Cell* **94**, 607–613 (1998).

6. S. Stoler, K. C. Keith, K. E. Curnick, M. Fitzgerald-Hayes, A mutation in CSE4, an essential gene encoding a novel chromatin-associated protein in yeast, causes chromosome nondisjunction and cell cycle arrest at mitosis. *Genes Dev.* **9**, 573–586 (1995).

7. M. Winey, C. L. Mamay, E. T. O'Toole, D. N. Mastronarde, T. H. Giddings, Jr., K. L. McDonald, J. R. McIntosh, Three-dimensional ultrastructural analysis of the Saccharomyces cerevisiae mitotic spindle. *J. Cell Biol.* **129**, 1601–1615 (1995).

8. S. Furuyama, S. Biggins, Centromere identity is specified by a single centromeric nucleosome in budding yeast. *Proc. Natl. Acad. Sci. U.S.A.* **104**, 14706–14711 (2007).

9. G. Cottarel, J. H. Shero, P. Hieter, J. H. Hegemann, A 125-base-pair CEN6 DNA fragment is sufficient for complete meiotic and mitotic centromere functions in Saccharomyces cerevisiae. *Mol. Cell. Biol.* **9**, 3342–3349 (1989).

10. H. A. Cole, B. H. Howard, D. J. Clark, The centromeric nucleosome of budding yeast is perfectly positioned and covers the entire centromere. *Proc. Natl. Acad. Sci. U.S.A.* **108**, 12687–12692 (2011).

11. K. Krassovsky, J. G. Henikoff, S. Henikoff, Tripartite organization of centromeric chromatin in budding yeast. *Proc. Natl. Acad. Sci. U.S.A.* **109**, 243–248 (2012).

12. S. Henikoff, J. G. Henikoff, "Point" centromeres of Saccharomyces harbor single centromere-specific nucleosomes. *Genetics* **190**, 1575–1577 (2012).

13. L. Clarke, J. Carbon, Isolation of a yeast centromere and construction of functional small circular chromosomes. *Nature* **287**, 504–509 (1980).

14. M. Fitzgerald-Hayes, L. Clarke, J. Carbon, Nucleotide sequence comparisons and functional analysis of yeast centromere DNAs. *Cell* **29**, 235–244 (1982).

15. L. Clarke, J. Carbon, Genomic substitutions of centromeres in Saccharomyces cerevisiae. *Nature* **305**, 23–28 (1983).

16. J. Mellor, W. Jiang, M. Funk, J. Rathjen, C. A. Barnes, T. Hinz, J. H. Hegemann, P. Philippsen, CPF1, a yeast protein which functions in centromeres and promoters. *EMBO J.* **9**, 4017–4026 (1990).

17. R. E. Baker, D. C. Masison, Isolation of the gene encoding the Saccharomyces cerevisiae centromere-binding protein CP1. *Mol. Cell. Biol.* **10**, 2458–2467 (1990).

18. M. Cai, R. W. Davis, Yeast centromere binding protein CBF1, of the helix-loop-helix protein family, is required for chromosome stability and methionine prototrophy. *Cell* **61**, 437–446 (1990).

19. J. Lechner, J. Carbon, A 240 kd multisubunit protein complex, CBF3, is a major component of the budding yeast centromere. *Cell* **64**, 717–725 (1991).

20. L. Panzeri, L. Landonio, A. Stotz, P. Philippsen, Role of conserved sequence elements in yeast centromere DNA. *EMBO J.* **4**, 1867–1874 (1985).

21. J. McGrew, B. Diehl, M. Fitzgerald-Hayes, Single base-pair mutations in centromere element III cause aberrant chromosome segregation in Saccharomyces cerevisiae. *Mol. Cell. Biol.* **6**, 530–538 (1986).

22. R. Ng, J. Carbon, Mutational and in vitro protein-binding studies on centromere DNA from Saccharomyces cerevisiae. *Mol. Cell. Biol.* **7**, 4522–4534 (1987).

23. J. H. Hegemann, J. H. Shero, G. Cottarel, P. Philippsen, P. Hieter, Mutational analysis of centromere DNA from chromosome VI of Saccharomyces cerevisiae. *Mol. Cell. Biol.* **8**, 2523–2535 (1988).

24. B. Jehn, R. Niedenthal, J. H. Hegemann, In vivo analysis of the Saccharomyces cerevisiae centromere CDEIII sequence: Requirements for mitotic chromosome segregation. *Mol. Cell. Biol.* **11**, 5212–5221 (1991).

25. K. F. Doheny, P. K. Sorger, A. A. Hyman, S. Tugendreich, F. Spencer, P. Hieter, Identification of essential components of the S. cerevisiae kinetochore. *Cell* **73**, 761–774 (1993).

26. P. Y. Goh, J. V. Kilmartin, NDC10: A gene involved in chromosome segregation in Saccharomyces cerevisiae. *J. Cell Biol.* **121**, 503–512 (1993).

27. R. Niedenthal, R. Stoll, J. H. Hegemann, In vivo characterization of the Saccharomyces cerevisiae centromere DNA element I, a binding site for the helix-loop-helix protein CPF1. *Mol. Cell. Biol.* **11**, 3545–3553 (1991).

28. S. Cumberledge, J. Carbon, Mutational analysis of meiotic and mitotic centromere function in Saccharomyces cerevisiae. *Genetics* **117**, 203–212 (1987).

29. A. Gaudet, M. Fitzgerald-Hayes, Mutations in CEN3 cause aberrant chromosome segregation during meiosis in Saccharomyces cerevisiae. *Genetics* **121**, 477–489 (1989).

30. M. R. Murphy, D. M. Fowlkes, M. Fitzgerald-Hayes, Analysis of centromere function in Saccharomyces cerevisiae using synthetic centromere mutants. *Chromosoma* **101**, 189–197 (1991).

31. J. Ortiz, O. Stemmann, S. Rank, J. Lechner, A putative protein complex consisting of Ctf19, Mcm21, and Okp1 represents a missing link in the budding yeast kinetochore. *Genes Dev.* **13**, 1140–1155 (1999).

32. T. Bechert, S. Heck, U. Fleig, S. Diekmann, J. H. Hegemann, All 16 centromere DNAs from Saccharomyces cerevisiae show DNA curvature. *Nucleic Acids Res.* **27**, 1444–1449 (1999).

33. H. S. Koo, H. M. Wu, D. M. Crothers, DNA bending at adenine . thymine tracts. *Nature* **320**, 501–506 (1986).

34. K. Yan, J. Yang, Z. Zhang, S. H. McLaughlin, L. Chang, D. Fasci, A. E. Ehrenhofer-Murray, A. J. R. Heck, D. Barford, Structure of the inner kinetochore CCAN complex assembled onto a centromeric nucleosome. *Nature* **574**, 278–282 (2019).

35. Z. Zhang, D. Bellini, D. Barford, Crystal structure of the Cenp-HIKHead-TW sub-module of the inner kinetochore CCAN complex. *Nucleic Acids Res.* **48**, 11172–11184 (2020).

36. R. Guan, T. Lian, B. R. Zhou, E. He, C. Wu, M. Singleton, Y. Bai, Structural and dynamic mechanisms of CBF3-guided centromeric nucleosome formation. *Nat. Commun.* **12**, 1763 (2021).

37. Y. Chen, R. E. Baker, K. C. Keith, K. Harris, S. Stoler, M. Fitzgerald-Hayes, The N terminus of the centromere H3-like protein Cse4p performs an essential function distinct from that of the histone fold domain. *Mol. Cell. Biol.* **20**, 7037–7048 (2000).

38. K. C. Keith, R. E. Baker, Y. Chen, K. Harris, S. Stoler, M. Fitzgerald-Hayes, Analysis of primary structural determinants that distinguish the centromere-specific function of histone variant Cse4p from histone H3. *Mol. Cell. Biol.* **19**, 6130–6139 (1999).

39. E. A. Anedchenko, A. Samel-Pommerencke, T. M. Tran Nguyen, S. Shahnejat-Bushehri, J. Popsel, D. Lauster, A. Herrmann, J. Rappsilber, A. Cuomo, T. Bonaldi, A. E. Ehrenhofer-Murray, The kinetochore module $^{\text{Okp1CENP-Q}}$/Ame1$^{\text{CENP-U}}$ is a reader for N-terminal modifications on the centromeric histone Cse4$^{\text{CENP-A}}$. *EMBO J.* **38**, e98991 (2019).

40. J. Fischböck-Halwachs, S. Singh, M. Potocnjak, G. Hagemann, V. Solis-Mezarino, S. Woike, M. Ghodgaonkar-Steger, F. Weissmann, L. D. Gallego, J. Rojas, J. Andreani, A. Kohler, F. Herzog, The COMA complex interacts with Cse4 and positions Sli15/Ipl1 at the budding yeast inner kinetochore. *eLife* **8**, e42879 (2019).

41. M. L. Dechassa, K. Wyns, M. Li, M. A. Hall, M. D. Wang, K. Luger, Structure and Scm3-mediated assembly of budding yeast centromeric nucleosomes. *Nat. Commun.* **2**, 313 (2011).

42. S. Yatskevich, K. W. Muir, D. Bellini, Z. Zhang, J. Yang, T. Tischer, M. Predin, T. Dendooven, S. H. McLaughlin, D. Barford, Structure of the human inner kinetochore bound to a centromeric CENP-A nucleosome. *Science* **376**, 844–852 (2022).

43. J. Lang, A. Barber, S. Biggins, An assay for de novo kinetochore assembly reveals a key role for the CENP-T pathway in budding yeast. *eLife* **7**, e37819 (2018).

44. S. J. Dowell, J. S. Tsang, J. Mellor, The centromere and promoter factor 1 of yeast contains a dimerisation domain located carboxy-terminal to the bHLH domain. *Nucleic Acids Res.* **20**, 4229–4236 (1992).

45. S. K. Nair, S. K. Burley, X-ray structures of Myc-Max and Mad-Max recognizing DNA. Molecular bases of regulation by proto-oncogenic transcription factors. *Cell* **112**, 193–205 (2003).

46. A. R. Ferre-D'Amare, G. C. Prendergast, E. B. Ziff, S. K. Burley, Recognition by Max of its cognate DNA through a dimeric b/HLH/Z domain. *Nature* **363**, 38–45 (1993).

47. D. C. Masison, K. F. O'Connell, R. E. Baker, Mutational analysis of the Saccharomyces cerevisiae general regulatory factor CP1. *Nucleic Acids Res.* **21**, 4133–4141 (1993).

48. P. K. Foreman, R. W. Davis, Point mutations that separate the role of Saccharomyces cerevisiae centromere binding factor 1 in chromosome segregation from its role in transcriptional activation. *Genetics* **135**, 287–296 (1993).

49. C. L. White, R. K. Suto, K. Luger, Structure of the yeast nucleosome core particle reveals fundamental changes in internucleosome interactions. *EMBO J.* **20**, 5207–5218 (2001).

50. K. Yan, Z. Zhang, J. Yang, S. H. McLaughlin, D. Barford, Architecture of the CBF3-centromere complex of the budding yeast kinetochore. *Nat. Struct. Mol. Biol.* **25**, 1103–1110 (2018).

51. W. Zhang, N. Lukoynova, S. Miah, J. Lucas, C. K. Vaughan, Insights into centromere DNA bending revealed by the Cryo-EM structure of the core centromere binding factor 3 with Ndc10. *Cell Rep.* **24**, 744–754 (2018).

52. V. Leber, A. Nans, M. R. Singleton, Structural basis for assembly of the CBF3 kinetochore complex. *EMBO J.* **37**, 269–281 (2018).

53. P. D. Lee, H. Wei, D. Tan, S. C. Harrison, Structure of the centromere binding factor 3 complex from Kluyveromyces lactis. *J. Mol. Biol.* **431**, 4444–4454 (2019).

54. R. L. Cohen, C. W. Espelin, P. De Wulf, P. K. Sorger, S. C. Harrison, K. T. Simons, Structural and functional dissection of Mif2p, a conserved DNA-binding kinetochore protein. *Mol. Biol. Cell* **19**, 4480–4491 (2008).

55. K. Tunyasuvunakool, J. Adler, Z. Wu, T. Green, M. Zielinski, Augustin Žídek, A. Bridgland, A. Cowie, C. Meyer, A. Laydon, S. Velankar, G. J. Kleywegt, A. Bateman, R. Evans, A. Pritzel, M. Figurnov, O. Ronneberger, R. Bates, S. A. A. Kohl, A. Potapenko, A. J. Ballard, B. Romera-Paredes, S. Nikolov, R. Jain, E. Clancy, D. Reiman, S. Petersen, A. W. Senior, K. Kavukcuoglu, E. Birney, P. Kohli, J. Jumper, D. Hassabis, Highly accurate protein structure prediction for the human proteome. *Nature* **596**, 590–596 (2021).

56. R. Camahort, B. Li, L. Florens, S. K. Swanson, M. P. Washburn, J. L. Gerton, Scm3 is essential to recruit the histone h3 variant cse4 to centromeres and to maintain a functional kinetochore. *Mol. Cell* **26**, 853–865 (2007).

57. K. Cieslinski, Y.-L. Wu, L. Nechyporenko, S. J. Hörner, D. Conti, M. Skruzny, J. Ries, Nanoscale structural organization and stoichiometry of the budding yeast kinetochore. *J. Cell Biol.* **222**, e202209094 (2023).

58. F. Spencer, P. Hieter, Centromere DNA mutations induce a mitotic delay in Saccharomyces cerevisiae. *Proc. Natl. Acad. Sci. U.S.A.* **89**, 8908–8912 (1992).

59. A. Gaudet, M. Fitzgerald-Hayes, Alterations in the adenine-plus-thymine-rich region of CEN3 affect centromere function in Saccharomyces cerevisiae. *Mol. Cell. Biol.* **7**, 68–75 (1987).

60. R. E. Baker, K. Rogers, Genetic and genomic analysis of the AT-rich centromere DNA element II of Saccharomyces cerevisiae. *Genetics* **171**, 1463–1475 (2005).

61. R. Perales, L. Zhang, D. Bentley, Histone occupancy in vivo at the 601 nucleosome binding element is determined by transcriptional history. *Mol. Cell. Biol.* **31**, 3485–3496 (2011).

62. A. R. Popchock, J. D. Larson, J. Dubrulle, C. L. Asbury, S. Biggins, Single molecule visualization of native centromeric nucleosome formation reveals coordinated deposition by kinetochore proteins and centromere DNA sequence. bioRxiv 2023.01.20.524981 [**Preprint**]. 21 January 2023. https://doi.org/10.1101/2023.01.20.524981.

63. G. E. Hamilton, L. A. Helgeson, C. L. Noland, C. L. Asbury, Y. N. Dimitrova, T. N. Davis, Reconstitution reveals two paths of force transmission through the kinetochore. *eLife* **9**, e56582 (2020).

64. J. Jumper, R. Evans, A. Pritzel, T. Green, M. Figurnov, O. Ronneberger, K. Tunyasuvunakool, R. Bates, A. Zidek, A. Potapenko, A. Bridgland, C. Meyer, S. A. A. Kohl, A. J. Ballard, A. Cowie, B. Romera-Paredes, S. Nikolov, R. Jain, J. Adler, T. Back, S. Petersen, D. Reiman, E. Clancy, M. Zielinski, M. Steinegger, M. Pacholska, T. Berghammer, S. Bodenstein, D. Silver, O. Vinyals, A. W. Senior, K. Kavukcuoglu, P. Kohli, D. Hassabis, Highly accurate protein structure prediction with AlphaFold. *Nature*, **596**, 583–589 (2021).

65. S. Deng, J. Cai, S. C. Harrison, H. Zhou, S. M. Hinshaw, Recognition of centromere-specific histone Cse4 by the inner kinetochore Okp1-Ame1 complex. bioRxiv 2023.06.06.543957 [**Preprint**]. 7 June 2023. https://doi.org/10.1101/2023.06.06.543957.

66. K. Dhatchinamoorthy, M. Shivaraju, J. J. Lange, B. Rubinstein, J. R. Unruh, B. D. Slaughter, J. L. Gerton, Structural plasticity of the living kinetochore. *J. Cell Biol.* **216**, 3551–3570 (2017).

67. A. Samel, A. Cuomo, T. Bonaldi, A. E. Ehrenhofer-Murray, Methylation of CenH3 arginine 37 regulates kinetochore integrity and chromosome segregation. *Proc. Natl. Acad. Sci. U.S.A.* **109**, 9029–9034 (2012).

68. R. Camahort, M. Shivaraju, M. Mattingly, B. Li, S. Nakanishi, D. Zhu, A. Shilatifard, J. L. Workman, J. L. Gerton, Cse4 is part of an octameric nucleosome in budding yeast. *Mol. Cell* **35**, 794–805 (2009).

69. P. Aravamudhan, I. Felzer-Kim, A. P. Joglekar, The budding yeast point centromere associates with two Cse4 molecules during mitosis. *Curr. Biol.* **23**, 770–774 (2013).

70. J. Wisniewski, B. Hajj, J. Chen, G. Mizuguchi, H. Xiao, D. Wei, M. Dahan, C. Wu, Imaging the fate of histone Cse4 reveals de novo replacement in S phase and subsequent stable residence at centromeres. *eLife* **3**, e02203 (2014).

71. A. P. Joglekar, D. C. Bouck, J. N. Molk, K. S. Bloom, E. D. Salmon, Molecular architecture of a kinetochore-microtubule attachment site. *Nat. Cell Biol.* **8**, 581–585 (2006).

72. J. Lawrimore, K. S. Bloom, E. D. Salmon, Point centromeres contain more than a single centromere-specific Cse4 (CENP-A) nucleosome. *J. Cell Biol.* **195**, 573–582 (2011).

73. M. Shivaraju, J. R. Unruh, B. D. Slaughter, M. Mattingly, J. Berman, J. L. Gerton, Cell-cycle-coupled structural oscillation of centromeric nucleosomes in yeast. *Cell* **150**, 304–316 (2012).

74. T. Gkikopoulos, V. Singh, K. Tsui, S. Awad, M. J. Renshaw, P. Scholfield, G. J. Barton, C. Nislow, T. U. Tanaka, T. Owen-Hughes, The SWI/SNF complex acts to constrain distribution of the centromeric histone variant Cse4. *EMBO J.* **30**, 1919–1927 (2011).

75. K. C. Scott, K. S. Bloom, Lessons learned from counting molecules: How to lure CENP-A into the kinetochore. *Open Biol.* **4**, 140191 (2014).

76. H. Xiao, G. Mizuguchi, J. Wisniewski, Y. Huang, D. Wei, C. Wu, Nonhistone Scm3 binds to AT-rich DNA to organize atypical centromeric nucleosome of budding yeast. *Mol. Cell* **43**, 369–380 (2011).

77. M. Funk, J. H. Hegemann, P. Philippsen, Chromatin digestion with restriction endonucleases reveals 150-160 bp of protected DNA in the centromere of chromosome XIV in Saccharomyces cerevisiae. *Mol. Gen. Genet.* **219**, 153–160 (1989).

78. A. Wilmen, J. H. Hegemann, The chromatin of the Saccharomyces cerevisiae centromere shows cell-type specific changes. *Chromosoma* **104**, 489–503 (1996).

79. K. S. Bloom, J. Carbon, Yeast centromere DNA is in a unique and highly ordered structure in chromosomes and small circular minichromosomes. *Cell* **29**, 305–317 (1982).

80. H. Xiao, F. Wang, J. Wisniewski, A. K. Shaytan, R. Ghirlando, P. C. FitzGerald, Y. Huang, D. Wei, S. Li, D. Landsman, A. R. Panchenko, C. Wu, Molecular basis of CENP-C association with the CENP-A nucleosome at yeast centromeres. *Genes Dev.* **31**, 1958–1972 (2017).

81. M. E. Pesenti, T. Raisch, D. Conti, K. Walstein, I. Hoffmann, D. Vogt, D. Prumbaum, I. R. Vetter, S. Raunser, A. Musacchio, Structure of the human inner kinetochore CCAN complex and its significance for human centromere organization. *Mol. Cell* **82**, 2113–2131.e8 (2022).

82. T. Tian, L. Chen, Z. Dou, Z. Yang, X. Gao, X. Yuan, C. Wang, R. Liu, Z. Shen, P. Gui, M. Teng, X. Meng, D. L. Hill, L. Li, X. Zhang, X. Liu, L. Sun, J. Zang, X. Yao, Structural insights into human CCAN complex assembled onto DNA. *Cell Discov.* **8**, 90 (2022).

83. K. Walstein, A. Petrovic, D. Pan, B. Hagemeier, D. Vogt, I. R. Vetter, A. Musacchio, Assembly principles and stoichiometry of a complete human kinetochore module. *Sci. Adv.* **7**, eabg1037 (2021

84. P. Hornung, P. Troc, F. Malvezzi, M. Maier, Z. Demianova, T. Zimniak, G. Litos, F. Lampert, A. Schleiffer, M. Brunner, K. Mechtler, F. Herzog, T. C. Marlovits, S. Westermann, A cooperative mechanism drives budding yeast kinetochore assembly downstream of CENP-A. *J. Cell Biol.* **206**, 509–524 (2014).

85. Z. Zhang, J. Yang, D. Barford, Recombinant expression and reconstitution of multiprotein complexes by the USER cloning method in the insect cell-baculovirus expression system. *Methods* **95**, 13–25 (2016).

86. K. M. Hyland, J. Kingsbury, D. Koshland, P. Hieter, Ctf19p: A novel kinetochore protein in Saccharomyces cerevisiae and a potential link between the kinetochore and mitotic spindle. *J. Cell Biol.* **145**, 15–28 (1999).

87. D. Kimanius, L. Dong, G. Sharov, T. Nakane, S. H. W. Scheres, New tools for automated cryo-EM single-particle analysis in RELION-4.0. *Biochem. J.* **478**, 4169–4185 (2021).

88. A. Rohou, N. Grigorieff, CTFFIND4: Fast and accurate defocus estimation from electron micrographs. *J. Struct. Biol.* **192**, 216–221 (2015).

89. T. Bepler, A. Morin, M. Rapp, J. Brasch, L. Shapiro, A. J. Noble, B. Berger, Positive-unlabeled convolutional neural networks for particle picking in cryo-electron micrographs. *Nat. Methods* **16**, 1153–1160 (2019).

90. M. Chen, S. J. Ludtke, Deep learning-based mixed-dimensional Gaussian mixture model for characterizing variability in cryo-EM. *Nat. Methods* **18**, 930–936 (2021).

91. P. Emsley, B. Lohkamp, W. G. Scott, K. Cowtan, Features and development of Coot. *Acta Crystallogr. D Biol. Crystallogr.* **66**, 486–501 (2010).

92. P. V. Afonine, R. W. Grosse-Kunstleve, N. Echols, J. J. Headd, N. W. Moriarty, M. Mustyakimov, T. C. Terwilliger, A. Urzhumtsev, P. H. Zwart, P. D. Adams, Towards automated crystallographic structure refinement with phenix.refine. *Acta Crystallogr. D Biol. Crystallogr.* **68**, 352–367 (2012).

93. T. D. Goddard, C. C. Huang, E. C. Meng, E. F. Pettersen, G. S. Couch, J. H. Morris, T. E. Ferrin, UCSF ChimeraX: Meeting modern challenges in visualization and analysis. *Protein Sci.* **27**, 14–25 (2018).

94. R. Evans, M. O'Neill, A. Pritzel, N. Antropova, A. Senior, T. Green, A. Žídek, R. Bates, S. Blackwell, J. Yim, O. Ronneberger, S. Bodenstein, M. Zielinski, A. Bridgland, A. Potapenko, A. Cowie, K. Tunyasuvunakool, R. Jain, E. Clancy, P. Kohli, J. Jumper, D. Hassabis, Protein complex prediction with AlphaFold-Multimer. bioRxiv 2021.10.04.463034 [**Preprint**]. 10 March 2022. https://doi.org/10.1101/2021.10.04.463034.

95. P. T. Lowary, J. Widom, Nucleosome packaging and nucleosome positioning of genomic DNA. *Proc. Natl. Acad. Sci. U.S.A.* **94**, 1183–1188 (1997).

96. A. M. Waterhouse, J. B. Procter, D. M. Martin, M. Clamp, G. J. Barton, Jalview Version 2—A multiple sequence alignment editor and analysis workbench. *Bioinformatics* **25**, 1189–1191 (2009).

97. G. Hagemann, V. Solis-Mezarino, S. Singh, M. Potocnjak, C. Kumar, F. Herzog, Quantitative crosslinking and mass spectrometry determine binding interfaces and affinities mediating kinetochore stabilization. bioRxiv 2022.03.31.486303 [**Preprint**]. 1 April 2022. https://doi.org/10.1101/2022.03.31.486303.

98. K. Klare, J. R. Weir, F. Basilico, T. Zimniak, L. Massimiliano, N. Ludwigs, F. Herzog, A. Musacchio, CENP-C is a blueprint for constitutive centromere-associated network assembly within human kinetochores. *J. Cell Biol.* **210**, 11–22 (2015).

99. S. Pentakota, K. Zhou, C. Smith, S. Maffini, A. Petrovic, G. P. Morgan, J. R. Weir, I. R. Vetter, A. Musacchio, K. Luger, Decoding the centromeric nucleosome through CENP-N. *eLife* **6**, e33442 (2017).

100. T. Tian, X. Li, Y. Liu, C. Wang, X. Liu, G. Bi, X. Zhang, X. Yao, Z. H. Zhou, J. Zang, Molecular basis for CENP-N recognition of CENP-A nucleosome on the human kinetochore. *Cell Res.* **28**, 374–378 (2018).

101. S. M. Hinshaw, S. C. Harrison, The structure of the Ctf19c/CCAN from budding yeast. *eLife* **8**, e44239 (2019).

102. V. Mariani, M. Biasini, A. Barbato, T. Schwede, lDDT: A local superposition-free score for comparing protein structures and models using distance difference tests. *Bioinformatics* **29**, 2722–2728 (2013).
